# Gut dysbiosis modulates hyperoxia-induced bronchopulmonary dysplasia by promoting EMT through activating TLR4/NF-κB pathway

**DOI:** 10.1186/s10020-026-01529-x

**Published:** 2026-06-16

**Authors:** Yaqin Yan, Shuling Liang, Sen Li, Shunv Xie, Xiaohui Wang, Huayan Zhang

**Affiliations:** 1 Division of Neonatology and Center for Newborn Care, Guangzhou Women and Children’s Medical Center, Guangzhou Medical University, Guangzhou, China; 2 Guangzhou Institute of Pediatrics, Guangzhou Women and Children’s Medical Center, State Key Laboratory of Respiratory Disease, Guangdong Basic Research Center of Excellence for Respiratory Medicine, Guangzhou Medical University, Guangzhou, China

**Keywords:** Bronchopulmonary dysplasia, Gut dysbiosis, TLR4/NF-κB pathway, Epithelial-mesenchymal transition, Fecal microbiota transplantation

## Abstract

**Background:**

Bronchopulmonary dysplasia (BPD) is a major cause of morbidity and mortality in premature infants. Although gut microbial dysbiosis is implicated in BPD pathogenesis, the underlying mechanisms are poorly defined. This study aims to elucidate the specific pathway through which gut dysbiosis drives BPD pathology and to identify potential therapeutic targets.

**Methods:**

The experimental BPD model was established by hyperoxia (FiO_2_ 85%) in neonatal mice from postnatal days 1 to 14. Pulmonary alveolarization and inflammation were analyzed at postnatal day 15. The modulatory role of gut microbiota was assessed using antibiotic-induced dysbiosis and fecal microbiota transplantation (FMT) from normoxic mice. Gut microbiome analysis was performed using 16S rRNA gene sequencing. The specific signaling pathway was investigated using a pharmacological inhibitor of TLR4. Furthermore, the molecular mechanisms were investigated through western blotting, real-time quantitative PCR, ELISA, and immunofluorescence.

**Results:**

Hyperoxia exposure induced impaired alveolarization, disrupted gut barrier integrity, and gut dysbiosis. These pathological changes were accompanied by elevated pulmonary inflammation, potent activation of the TLR4/NF-κB pathway, and upregulation of epithelial-mesenchymal transition (EMT) associated markers. These changes were exacerbated by early postnatal antibiotic administration, whereas FMT from normoxic mice rescued these phenotypes, restored gut barrier function, suppressed TLR4/NF-κB signaling, and reversed EMT progression. Notably, pharmacological inhibition of TLR4 mirrored the protective effects of FMT, effectively attenuating hyperoxia-induced lung injury and EMT.

**Conclusions:**

Our findings establish a mechanistic link for the gut-lung axis in BPD, demonstrating that gut dysbiosis is a critical modulator of lung development impairment and pathological EMT via activation of the TLR4/NF-κB pathway.

**Supplementary Information:**

The online version contains supplementary material available at 10.1186/s10020-026-01529-x.

## Introduction

Bronchopulmonary dysplasia (BPD), affecting approximately 45% of infants born at < 29 weeks’ gestational age, is one of the most common complications in premature infants (Gilfillan et al. 2021). Despite the recent advancements in the field of neonatology, the incidence of BPD has been increasing due to improved survival of extremely premature infants (Thébaud et al. 2019). BPD is typically characterized by alveolar simplification, damaged microvascular development and abnormal pulmonary function (Gilfillan et al. 2021). Unfortunately, the multifactorial factors contributing to the pathogenesis of BPD have been incompletely understood, hindering the development of effective preventive or therapeutic strategies.

Increasing evidence supports a functional gut-lung axis, whereby the gut microbiota influences pulmonary development and disease (Özçam and Lynch 2024). Severe BPD has been associated with gut microbial dysbiosis, characterized by reduced microbial diversity, depletion of anti-inflammatory taxa such as *Lactobacillus*, and enrichment of pro-inflammatory and potentially pathogenic bacteria like *Proteobacteria* in BPD patients (Gao et al. 2024; Yang et al. 2021; Gallacher et al. 2020; Ryan et al. 2019; Zhang et al. 2022; Zhu and Ding 2025). Germ-free neonatal mice exposed to hyperoxia exhibit attenuated alveolar hypoplasia and pulmonary inflammation compared with conventionally colonized counterparts, underscoring a contributory role of gut microbiota in hyperoxia-induced BPD (Dolma et al. 2020). Despite these associations, the mechanisms by which gut microbial dysbiosis drives BPD pathogenesis remain poorly defined.

Accumulating evidence indicates that gut microbiota is a key regulator of pulmonary immune homeostasis (Özçam and Lynch 2024; Budden et al. 2017). Given the central role of inflammation in the pathogenesis of BPD, disruption of the gut microbial ecosystem may influence disease severity by modulating lung inflammatory response (Gao et al. 2024; Yang et al. 2021). Additionally, epithelial-mesenchymal transition (EMT) is a well-characterized pathogenic phenomenon observed in hyperoxia‐induced BPD model (Hua et al. 2025; Yang et al. 2014; Hou et al. 2015). We therefore hypothesized that gut microbial dysbiosis-induced intestinal barrier dysfunction amplified pulmonary inflammation and promoted epithelial-mesenchymal transition through activation of the TLR4/NF-κB signaling pathway, thereby exacerbating hyperoxia-induced BPD. To test this hypothesis, we employed antibiotic-mediated microbiota disruption and fecal microbiota transplantation (FMT) in a murine model of BPD. These approaches allowed us to determine whether manipulating gut microbiome modulates BPD development and to explore the underlying mechanisms driving these processes.

## Materials and methods

### Animals

According to the well-established murine model of BPD (Sucre et al. 2020), neonatal mice with their mothers were randomly divided into hyperoxia (FiO_2_ 85%, HA) or room air (FiO_2_ 21%, RA) within 12 h after birth for 2 weeks. The nursing mothers were rotated between hyperoxia and room air groups every 24 h to avoid oxygen toxicity. All groups have free access to food and water. The mice were exposed to oxygen in a modified controller (Yuyan, China) and oxygen levels were maintained continuously using the monitor. This study was approved by the Institutional Animal Care and Use Committee of Guangzhou Women and Children's Medical Center (KTDW-2024–00973). Mice were euthanized on days 15 by inhaled isoflurane, and then tissues were harvested for subsequent experiments.

To investigate whether the BPD pathogenesis was linked to the gut microbiota, a subgroup of the hyperoxia-exposed mice received daily gavage administration of broad-spectrum antibiotics (100 mg/kg neomycin sulfate and 50 mg/kg vancomycin, Sigma) from postnatal day (PN) 1 to PN14 to disturb gut microbiota. The control group was administrated with PBS every day.

To further explore the roles of gut microbiota in the BPD pathogenesis, FMT was performed. Briefly, fecal material was obtained from RA mice. 80 ~ 110 mg of collected feces was homogenized in 1 ml of sterile saline and centrifuged for 5 min at 15,000 g to precipitate the microbiome. Bacterial pellets were resuspended in 200 ul of DPBS. Transfer 1 ul of the fecal microbiota suspension into a cuvette, measure its OD600 using a UV spectrophotometer, and adjust it to 1 ± 0.05, which corresponds to a bacterial concentration of approximately 10^^^8–10^^^9 cfu/ml. Then, hyperoxia-exposed mice were treated daily with fecal microbiota transplants from RA donor group via oral gavage at a ratio of 10 ul per gram from PN7 to PN14.

To evaluate the effect of TLR4/NF-κB signaling pathway in the BPD pathogenesis, the hyperoxia-exposed mice were injected subcutaneously with the TLR4 inhibitor TAK-242 (Resatorvid, 3 mg/kg/day) or vehicle alone from PN7 to PN 14.

### LPS and cytokines measurement

The concentrations of LPS, tumor necrosis factor-α (TNF-α), interleukin-1β (IL-1β), and interleukin-1β (IL-6) were determined using enzyme linked immunosorbent assay (ELISA) kits (Jingmei Biotechnology, China) according to the manufacturer’s instructions.

### Gut permeability analysis

Gut permeability was evaluated by measuring the serum level of fluorescein isothiocyanate conjugated-dextran (FD-4, Sigma, USA). Briefly, 4 h-fasted mice were gavage with FD-4 (0.1 ml/10 g, 50 mg/ml). After 4 h, the blood was collected after euthanasia. Then, serum was diluted in an equal volume of PBS and FD-4 concentrations in serum were detected by a fluorescence spectrophotometer (485 nm excitation and 528 nm emission wavelength). The serially diluted samples were taken as standards, and all the detected samples were analyzed in triplicate.

### Western blotting

Lung tissues were homogenized using RIPA buffer with added protease and phosphate inhibitors. Immunoblotting was performed with primary antibodies against Claudin1 (ABclonal, A21971, 1:2000), Occludin (ABclonal, A24601, 1:3000), ZO1 (HUABIO, HA722797, 1:1000), TLR4 (HUABIO, RT1666, 1:1000), NF-κB (Zenbio, 380,172, 1:1000), p-NF-κB (Zenbio, 310,013, 1:1000), SFTPC (ABclonal, A23181, 1:1000), AQP5 (ABclonal, A9927, 1:1000), α-SMA (ABclonal, A2235, 1:1000), β-Actin (ABclonal, AC026, 1:100,000), and HSP90α/β (ABclonal, A5027, 1:1000), and then incubated with HRP Goat Anti-Rabbit/Mouse IgG secondary antibody (ABclonal, AS014/AS003, 1:5000). Imaging was detected using a highly sensitive ECL chemiluminescence reagent (Abbkine, BMU101). Image J software was used to calculate the optical density of protein bands and then normalized to that of β-actin or HSP90α/β.

### Histopathological analysis

The left lungs were inflated and fixed with 4% paraformaldehyde (G1101, Servicebio, China) via tracheal intubation at a pressure of 25 cm H2O for 15 min. Then, the left lungs were removed and fixed in 4% paraformaldehyde. The lung tissues were cut into 5-μm-thick sections and stained with hematoxylin and eosin (HE), then examined under light microscopy. As previously described (Chen et al. 2023), the mean linear intercept (MLI), an indicator of the mean alveolar diameter, was assessed in 10 nonoverlapping fields. And the radial alveolar counts (RAC), an indicator of the maturity of developing lungs, refers to the number of alveoli on the vertical line from the center of the respiratory bronchioles to the nearest fibrous septum (or pleura) (Emery and Mithal 1960).

### Immunofluorescence staining

Lung tissue sections were deparaffinized and rehydrated, antigen repaired and blocked. Then sections were incubated with anti-SPC (1:200, ABclonal), anti-α-SMA (1:100, ABclonal), and anti-N-cadherin (1:200, HUABIO) at 4 °C overnight followed by secondary antibodies (1:200, FITC, MCE; 1:1000, Alexa Fluor 488, Abcam; 1:1000, Alexa Fluor 594, Abcam), and then stained with DAPI (Servicebio, Wuhan, China) for cell nuclei. Immunofluorescence images were acquired using a Leica DMi8 fluorescence microscope.

### 16S rRNA gene sequencing

Microbial genomic DNA was isolated from colonic contents using QIAamp DNA stool Mini Kit (Qiagen) as instructed by the manufacturer. The variable regions V3 and V4 of the 16S rRNA were amplified with primers 338 F (5′ ACTCCTACGGGAGGCAGCA 3′) and 806R (5′ GGACTACHVGGGTWTCTAAT 3′) and sequenced on the NovaSeq platform (Illumina) according to protocols. The microbial community analysis was conducted as previously reported. We thank Biomarker Technologie Co., Ltd for assisting in sequencing and bioinformatics analysis.

### Bacterial DNA quantification

Bacterial DNA was quantified via qPCR targeting the 16S rRNA gene with primers 338 F (5′ ACTCCTACGGGAGGCAGCA 3′) and 806R (5′ GGACTACHVGGGTWTCTAAT 3′). A standard curve was generated using serial dilutions of a plasmid containing a cloned 16S fragment, then plotting the Ct values against the logarithm of the known plasmid copy numbers. The bacterial load in fecal DNA samples was calculated from the standard curve and expressed as 16S rRNA gene copies per gram of feces.

### Quantitative real-time PCR

Total RNA was extracted from colon samples using *RNAiso Easy* following the manufacturer’s instructions (Takara, TCH021, China), and then reversely transcribed using *PrimeScript™ RT reagent Kit* (Takara, RR037A, China) to obtain cDNA. For quantification, PCR was performed on QuantStudio 6 Flex (Applied Biosystems) with SYBR™ Green (Thermo, A57156). Gene expression was quantified by the 2^−ΔΔ^CT method and used 18S as a reference gene. The primers applied in this study are listed in Table S1.

### Statistical analysis

All data were presented as mean ± SEM. Statistical analysis was performed by GraphPad Prism 8 and *P* < 0.05 was regarded as statistical significance. Data distribution and variance were examined prior to selecting appropriate tests. Student’s *t* test (when normal distribution) or Mann–Whitney U test (when non-normal distribution) was used to assess the significance between two groups. For comparisons involving three or more groups, one-way ANOVA followed by Tukey’s post hoc test was applied when data followed a normal distribution. When normality assumptions were violated, the non-parametric Kruskal–Wallis test with Dunn’s correction for multiple comparisons was used instead.

## Results

### Hyperoxia impaired lung development and weakened gut barrier function in neonatal mice

**Fig. 1:**
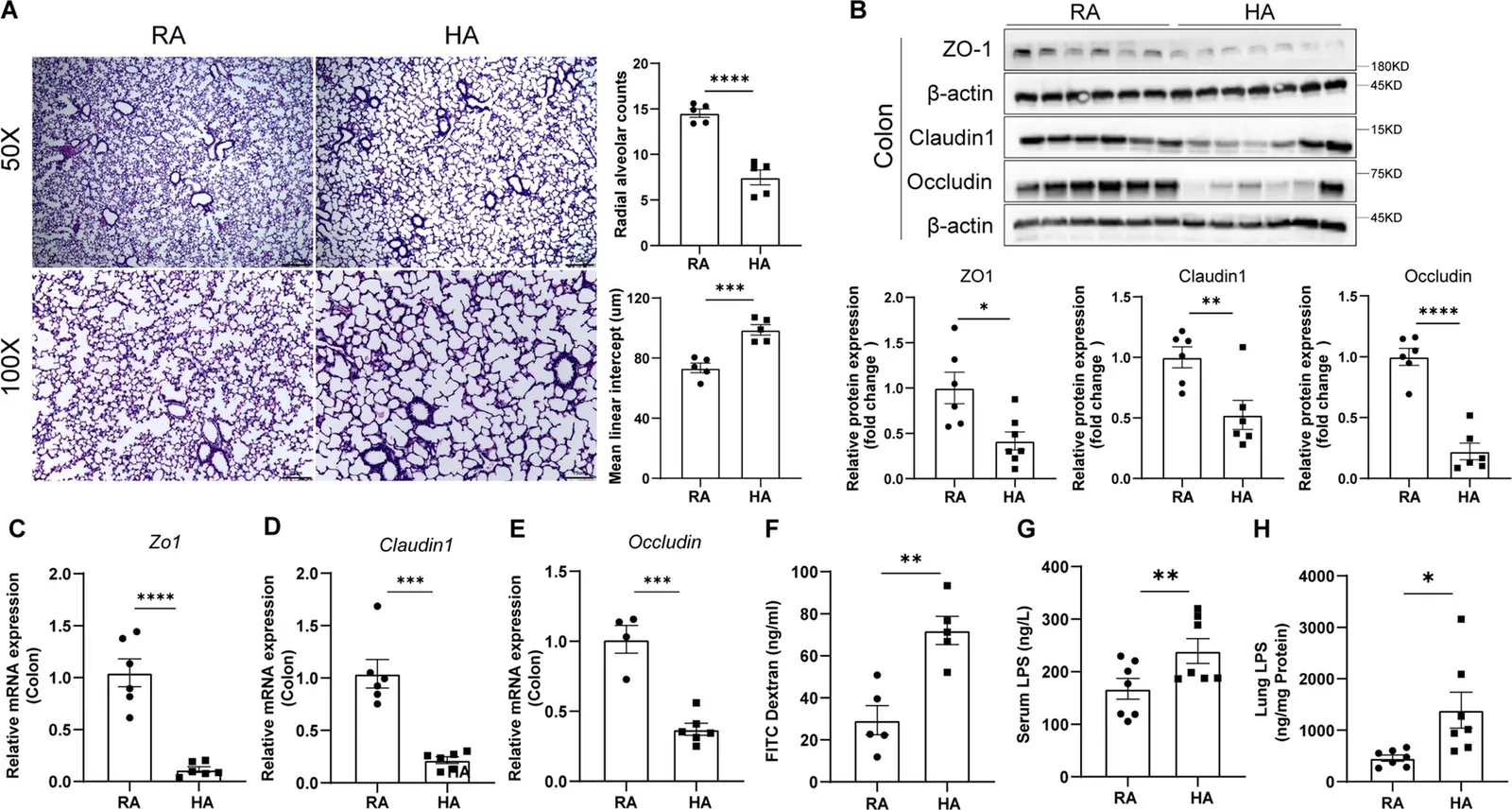
Hyperoxia impaired lung development and weakened gut barrier function in neonatal mice. **A** Representative lung histology, RAC and MLI from neonatal mice exposed to RA or hyperoxia (scale bar, 250 μm at magnification × 50 and 75 μm at magnification × 100). **B** Representative western blots and quantification of colonic ZO-1, Claudin1, and Occludin in RA and HA mice. **C-E** The mRNA expression of colonic *Zo-1*, *Claudin-1*, and *Occludin* in RA and HA mice. **F** Serum levels of FITC-dextran in RA and HA mice. **G** Serum levels of lipopolysaccharide (LPS) were measured by ELISA in RA and HA mice. **H** LPS levels in the lung tissues of RA and HA mice. Data were presented as mean ± SEM and representative of *n* = 5–7 mice per experimental group. Data are analyzed using Student’s *t*-test (**A-C**, **E**, **F**, **H**) or Mann–Whitney U test (**D**, **G**). ^*^P < 0.05, ^**^P < 0.01, ^***^P < 0.001, and ^****^P < 0.0001 vs. the RA group

The experimental BPD murine model was established by postnatal hyperoxia in neonatal mice. As evident by decreased radial alveolar count (RAC) and increased mean linear intercept (MLI), hyperoxia significantly reduced pulmonary alveolarization compared to the room air (RA) group (Fig. 1A). We next explored the effects of hyperoxia on gut barrier function. As shown in Fig. 1B, the levels of tight junction proteins including ZO1, Claudin-1 and Occludin were dramatically reduced in colonic tissue of the hyperoxia-exposed (HA) mice than those of RA mice. In addition, real-time qPCR analysis confirmed that the colonic mRNA expressions of *Zo1*, *Claudin-1* and *Occludin* were lower in HA group (Fig. 1C-E). Consistent with the disruption of barrier integrity, HA mice exhibited significantly increased gut permeability, evidenced by elevated serum levels of fluorescein isothiocyanate conjugated (FITC)-dextran following oral administration (Fig. 1F). Accordingly, the levels of LPS in the serum and lung tissues were also higher in HA mice (Fig. 1G, H). Together, these findings indicated that hyperoxia not only injured the developing lung but also compromised the gut barrier, leading to increased systemic and pulmonary endotoxin exposure.

### Early postnatal administration of antibiotics exacerbated both abnormal pulmonary development and gut barrier dysfunction in hyperoxia-exposed mice

**Fig. 2:**
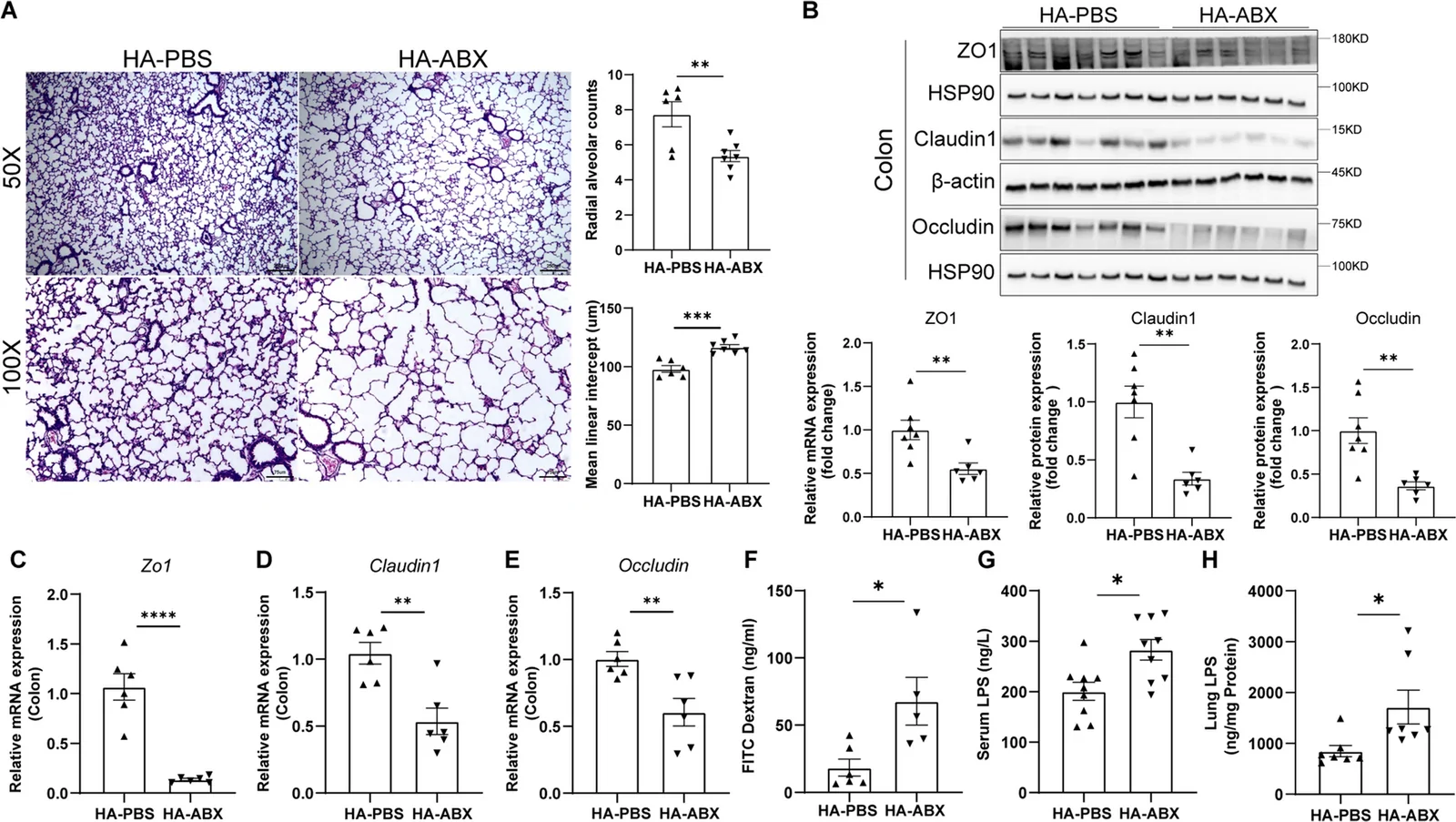
Early postnatal administration of antibiotics exacerbated both abnormal pulmonary development and gut barrier dysfunction in hyperoxia-exposed mice. **A** Representative lung histology, RAC and MLI in HA-PBS and HA-ABX mice (scale bar, 250 μm and 75 μm). **B** Representative western blots and quantification of colonic ZO-1, Claudin1, and Occludin in HA-PBS and HA-ABX mice. **C-E** The mRNA expression of colonic *Zo-1*, *Claudin-1*, and *Occludin* in HA-PBS and HA-ABX mice. **F** Serum levels of FITC-dextran in HA-PBS and HA-ABX mice. **G** Serum levels of LPS in HA-PBS and HA-ABX mice. **H** LPS levels in the lung tissues of HA-PBS and HA-ABX mice. Data were presented as mean ± SEM and representative of *n* = 6–9 mice per experimental group. Data are analyzed using Student’s *t*-test (**A-F**, **H**) or Mann–Whitney U test (**G**). ^*^P < 0.05, ^**^P < 0.01, ^***^P < 0.001, and ^****^P < 0.0001 vs. the HA-PBS group

As shown in Fig. 2A, HE staining demonstrated that antibiotics (ABX)-treated mice exposed to hyperoxia (HA-ABX) had more pronounced lung development impairment. These mice showed a marked decrease in the number of alveoli and enlargement of the alveolar space compared to PBS-treated hyperoxia-exposed mice (HA-PBS). However, ABX treatment failed to impair lung development in RA mice (Fig. S1A-C). When ABX were added, the hyperoxia-exposed mice showed reduced expression of epithelial tight junction genes including ZO1, Claudin-1 and Occludin (Fig. 2B-E). Furthermore, hyperoxia-exposed mice exhibited a marked elevation in serum levels of FITC-labeled dextran following ABX treatment (Fig. 2F). We also found that the levels of LPS in serum and lung tissues were increased in the HA-ABX mice compared with HA-PBS mice (Fig. 2G, H). These results implied that while antibiotics alone did not impair lung development under normoxia, they rendered the developing lung more susceptible to hyperoxia-induced injury, likely by disrupting gut barrier function.

### Hyperoxia and early-postnatal ABX treatment induced gut dysbiosis in neonatal mice

**Fig. 3:**
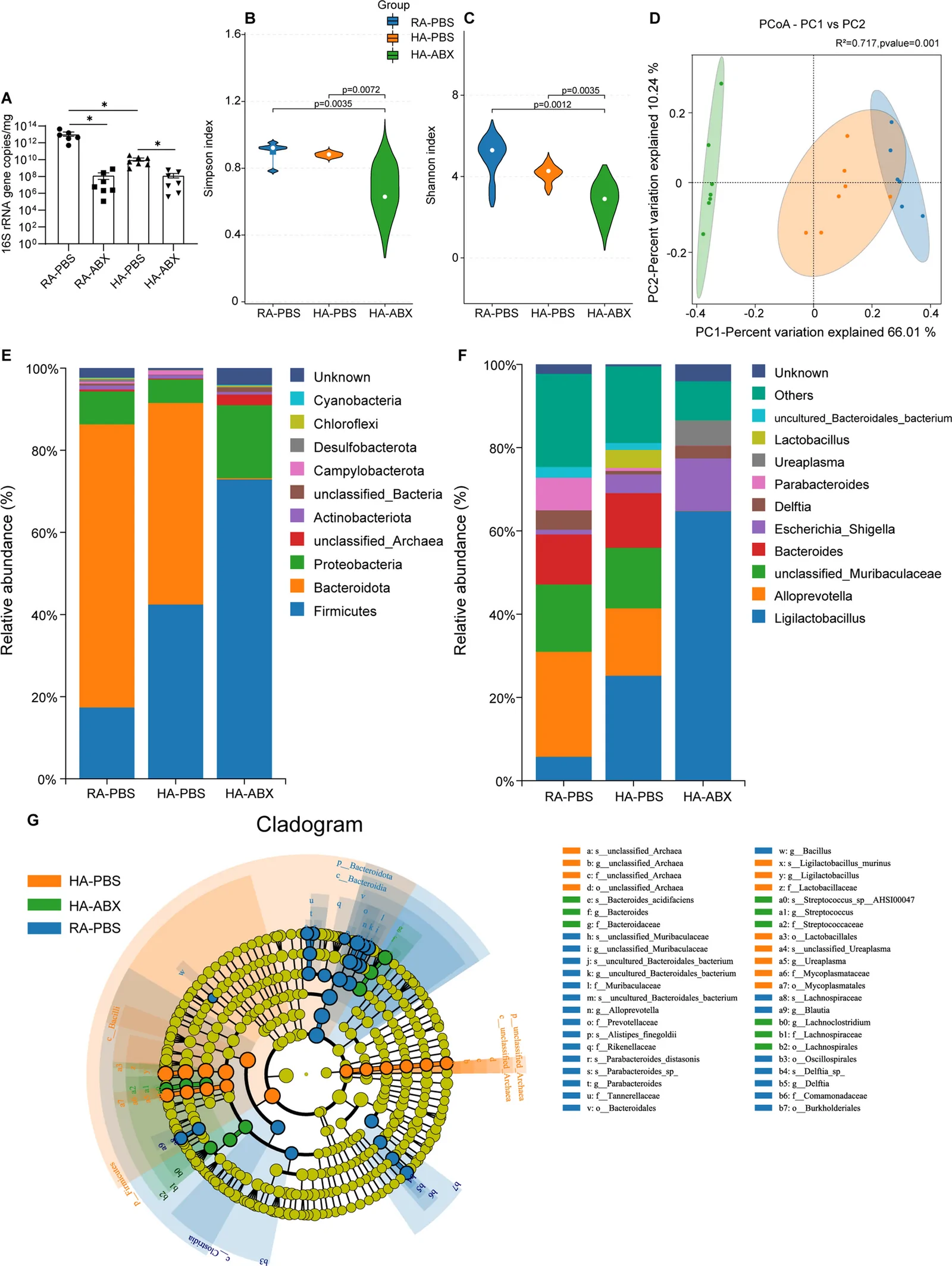
Hyperoxia and early-postnatal ABX treatment induced gut dysbiosis in neonatal mice. **A** Quantification of bacterial abundance via 16S rRNA-based quantitative PCR. **B-C** Comparison of Simpson and Shannon of α-diversity index. **D** PCoA based on weighted UniFrac distances. **E** Bacterial composition at the phylum level. **F** Bacterial composition at genus level. **G** Cladogram for differentially abundant taxa from LEfSe analysis. Data were presented as mean ± SEM and representative of *n* = 6–7 mice per experimental group. ^*^P < 0.05

Both hyperoxia exposure and early-postnatal ABX treatment reduced the abundance of gut microbiome (Fig. 3A). By employing high-throughput sequencing of 16S rRNA in colonic contents, our analysis revealed no notable differences in α-diversity between RA-PBS and HA-PBS groups. However, ABX treatment led to a significant reduction in α-diversity (Fig. 3B, C). Principal coordinate analysis (PCoA) showed that β-diversity clearly distinguished RA-PBS mice from HA-PBS mice, and ABX treatment caused even greater differences compared to HA-PBS mice (Fig. 3D), indicating that hyperoxia exposure and ABX treatment significantly altered the overall structure of the gut microbial community.

The overall composition of gut microbiota was further analyzed. At the phylum level, hyperoxia exposure resulted in a significant increase in the relative abundance of *Firmicutes*, concurrent with a decrease in *Bacteroidota*. These effects were further exacerbated by ABX treatment (Fig. 3E). The relative abundance of *Proteobacteria* was slightly decreased in HA-PBS mice but showed a marked increase following ABX treatment (Fig. 3E). At the genus level, hyperoxia and ABX treatment significantly increased the relative abundance of *Ligilactobacillus* and *Escherichia_Shigella* in the intestine. Conversely, these treatments resulted in a notable decline in the levels of *Alloprevotella*, *Bacteroides*, and *Parabacteroides* in the intestine when compared with the RA-PBS groups (Fig. 3F). LEfSe analysis revealed notable taxonomic differences among RA-PBS, HA-PBS and HA-ABX mice (Fig. 3G). Together, these data demonstrated that hyperoxia induced gut dysbiosis in neonatal mice, characterized by shifts in community structure and specific taxa, and that early-postnatal ABX treatment profoundly exacerbated these alterations.

### Early-postnatal ABX treatment promoted the TLR4/NF-κB signaling overactivation and epithelial-mesenchymal transition (EMT) in the lung of hyperoxia-exposed mice

**Fig. 4:**
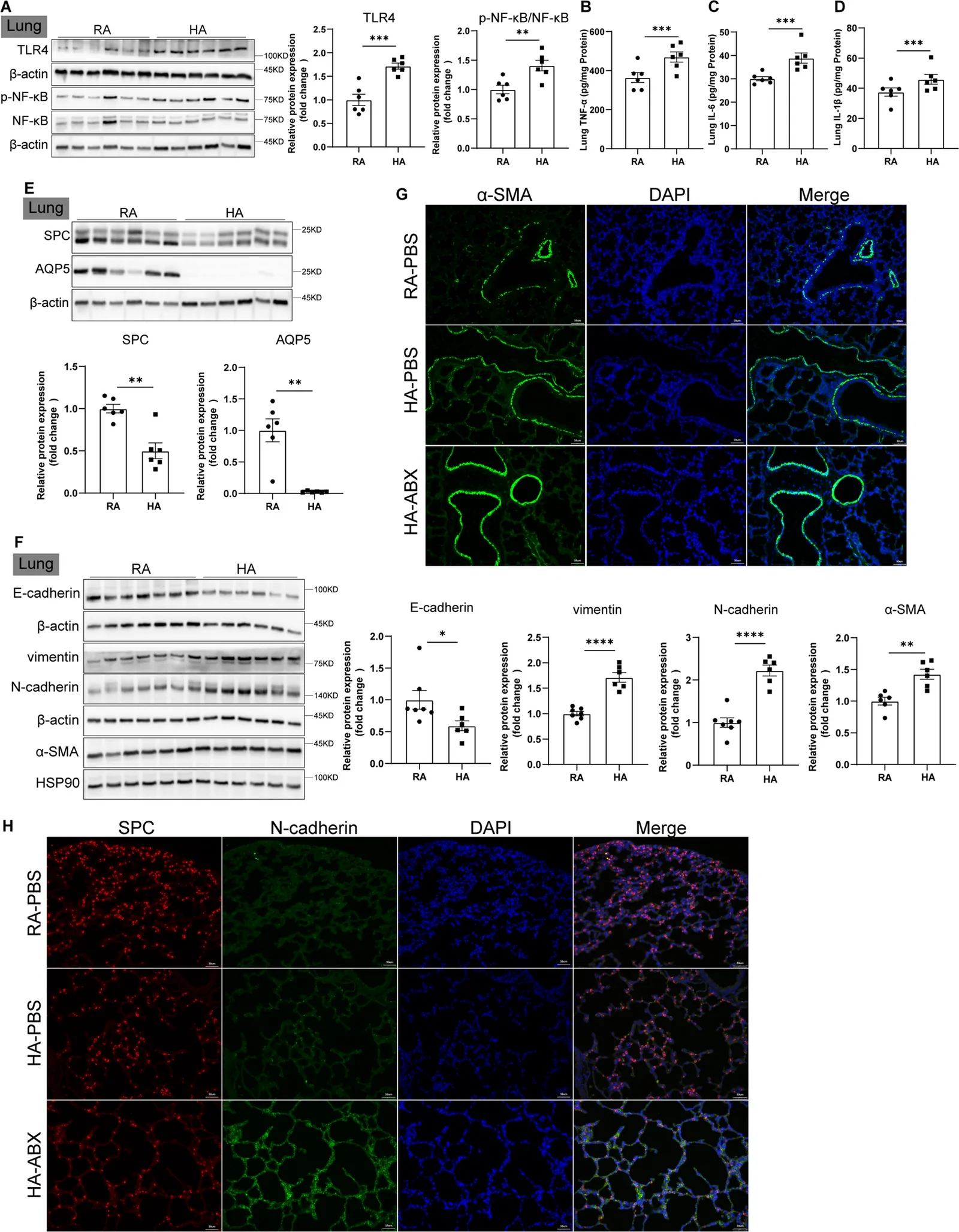
Activated TLR4/NF-κB signaling and EMT in the lung of hyperoxia-exposed mice. **A** Representative western blots and quantification of pulmonary TLR4 and p-NF-κB p65 in RA and HA mice. **B** TNF-α levels in the lung. **C** IL-6 levels in the lung. **D** IL-1β levels in the lung. **E** Representative western blots and quantification of pulmonary SPC and AQP5 in RA and HA mice. **F** Immunoblot analysis of E-cadherin, vimentin, N-cadherin, and α-SMA in lung tissues of RA and HA mice. **G** Immunofluorescence detection of α-SMA in lung tissues of RA-PBS group, HA-PBS group and HA-ABX group. Scale bar, 50 μm. **H** Immunofluorescence detection of SP-C and N-cadherin in lung tissues of RA-PBS group, HA-PBS group and HA-ABX group. Scale bar, 50 μm. Data were presented as mean ± SEM and representative of *n* = 4–7 mice per experimental group. Data are analyzed using Student’s *t*-test (**A-D**, **F**) or Mann–Whitney U test (**E**). ^*^P < 0.05, ^**^P < 0.01, ^***^P < 0.001, and ^****^P < 0.0001 vs. the RA group

It is well-established that TLR4 specifically recognizes the immune-stimulatory microbial ligand LPS, triggering NF-κB activation and subsequent proinflammatory signaling cascades (Luo et al. 2025; Tang et al. 2021). In the lungs of hyperoxia-exposed mice, western blot analysis revealed a marked upregulation of TLR4 and NF-κB phosphorylation, indicative of pathway activation (Fig. 4A). ABX treatment further augmented this hyperoxia-induced TLR4/NF-κB signaling activation (Fig. S2A). Consistently, hyperoxia exposure resulted in elevated levels of the pro-inflammatory cytokines TNF-α, IL-6, and IL-1β in lung homogenates (Fig. 4B-D), which were further increased by ABX treatment (Fig. S2B-D).

In the lung tissue of hyperoxia-exposed BPD mice, the levels of surfactant protein C (SP-C), a marker for alveolar epithelial type II cells (AECII), and aquaporin 5 (AQP5), a marker for AECI cells, were significantly lower than those in the RA group (Fig. 4E). This suggests that hyperoxia may impair the differentiation of AECII to AECI. Furthermore, mice exposed to hyperoxia demonstrated a markedly increased EMT, as evidenced by reduced E-cadherin expression and elevated levels of vimentin, N-cadherin, and α-SMA (Fig. 4F). ABX treatment further reduced the expression of SP-C and AQP5, exacerbating the enhanced EMT observed in the hyperoxia-exposed mice (Fig. S2E, F). In addition, immunofluorescence staining revealed increased α‐SMA expression in mice after hyperoxia and early-postnatal ABX treatment (Fig. 4G). As shown in immunofluorescence double staining, these treatments reduced SPC expression, while elevating N-cadherin expression (Fig. 4H). Collectively, these findings demonstrated that early-postnatal ABX treatment worsened lung development impairment in BPD possibly by promoting pulmonary inflammation and EMT.

### Transplantation of gut microbiota from RA mice ameliorated hyperoxia-induced BPD phenotype and restored gut barrier function

To further understand the role of gut-lung axis in BPD pathogenesis, we performed fecal microbiota transplantation (FMT). 16S rRNA sequencing was performed as shown in Fig. S3. We observed no significant differences in α-diversity among groups (Fig. S3A, B). PCoA based on weighted UniFrac revealed that β-diversity was highly similar between the HA-FMT and RA-PBS groups (Fig. S3C). Besides, taxonomic composition at the phylum and genus levels showed that the relative abundances of key taxa in HA-FMT mice resembled those in the RA-PBS group (Fig. S3D, E).

**Fig. 5:**
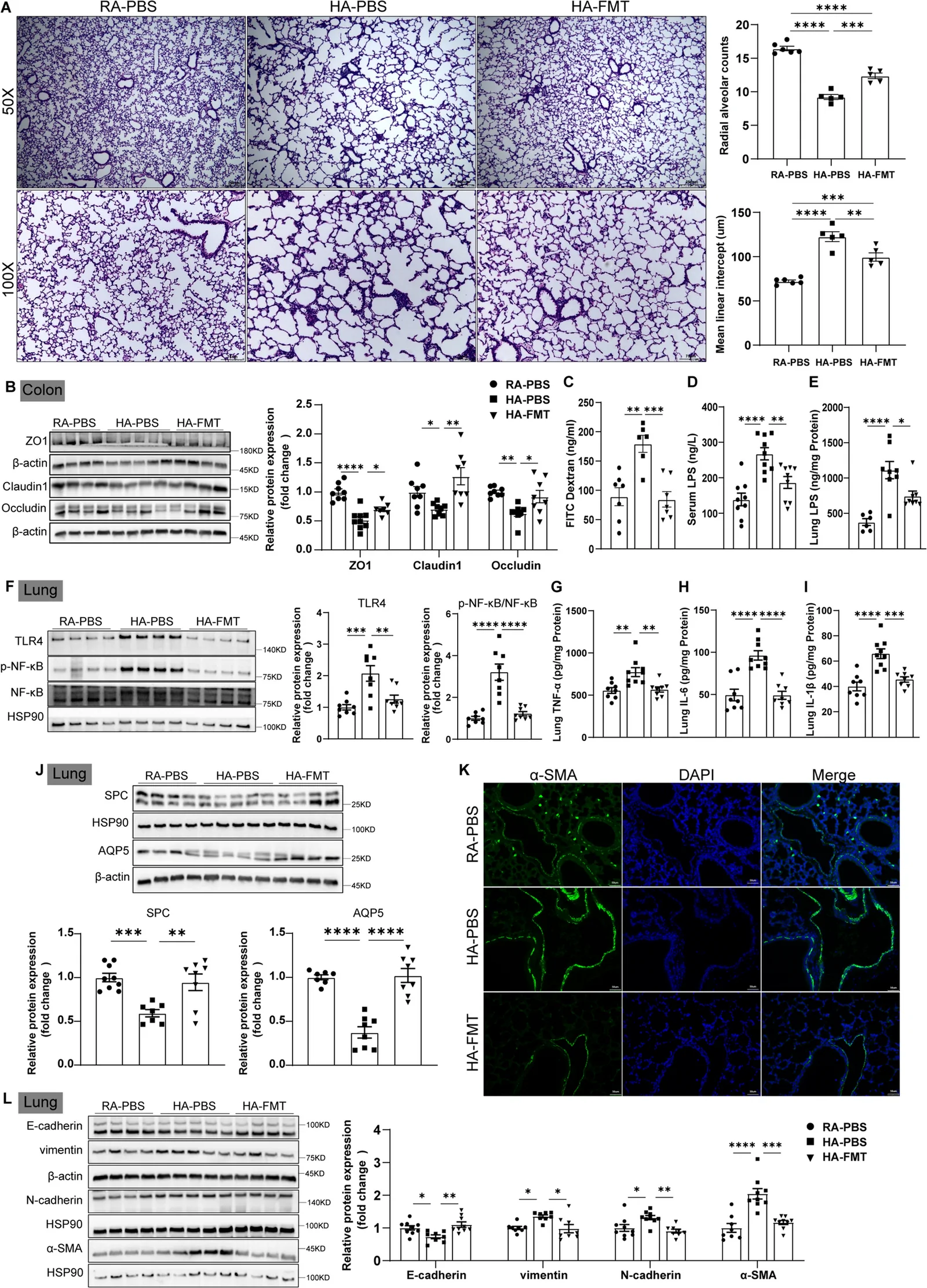
Transplantation of gut microbiota of RA mice ameliorated hyperoxia-induced BPD phenotype, restored gut barrier function, suppressed pulmonary TLR4/NF-κB signaling and inhibited EMT. **A** Representative lung histology, RAC and MLI in RA-PBS, HA-PBS and HA-FMT mice (scale bar, 250 μm and 75 μm). **B** Immunoblot analysis of ZO1, Claudin1, and Occludin in colon tissues of RA-PBS, HA-PBS and HA-FMT mice. **C** Serum levels of FITC-dextran in RA-PBS, HA-PBS and HA-FMT mice. **D** Serum levels of LPS in RA-PBS, HA-PBS and HA-FMT mice. **E** LPS levels in the lung tissues of RA-PBS, HA-PBS and HA-FMT mice. **F** Representative western blots and quantification of pulmonary TLR4 and p-NF-κB p65 in RA-PBS, HA-PBS and HA-FMT mice. **G** TNF-α levels in the lung. **H** IL-6 levels in the lung. **I** IL-1β levels in the lung. **J** Immunoblot analysis of SPC and AQP5 in lung tissues of RA-PBS, HA-PBS and HA-FMT mice. **K** Immunofluorescence detection of α-SMA in lung tissues of RA-PBS group, HA-PBS group and HA-FMT group. Scale bar, 50 μm. **L** Immunoblot analysis of E-cadherin, vimentin, N-cadherin, and α-SMA in lung tissues of RA-PBS group, HA-PBS group and HA-FMT group. Data were presented as mean ± SEM and representative of *n* = 4–9 mice per experimental group. Data are analyzed using one-way ANOVA with Tukey’s post hoc comparison (**A-D**, **F-J**, **L**) or Kruskal–Wallis with Dunn’s multiple comparisons test (**E**). ^*^P < 0.05, ^**^P < 0.01, ^***^P < 0.001, and.^****^P < 0.0001

As shown in Fig. 5A, HA mice receiving FMT from RA mice exhibited improved alveolarization, as demonstrated by a significant increase in RAC and a corresponding decrease in MLI. Furthermore, the hyperoxia-induced disruption of gut barrier function was attenuated following FMT, as evidenced by the upregulated colonic protein levels of ZO-1, Claudin-1 and Occludin, accompanied by a significant reduction in serum levels of FITC-labeled dextran (Fig. 5B, C). Accordingly, FMT with RA feces significantly reduced LPS levels in both the serum and lung tissues of mice exposed to hyperoxia (Fig. 5D, E). These findings highlighted a pivotal role of gut microbiota in mediating lung development in hyperoxia-exposed mice.

### Transplantation of gut microbiota from RA mice suppressed pulmonary EMT in hyperoxia-exposed mice, an effect associated with modulation of TLR4/NF-κB signaling

We next investigated how gut microbiota affects BPD. Based on the preceding findings, we hypothesized that gut dysbiosis contributes to impaired lung development, potentially by activating the TLR4/NF-κB pathway and promoting pulmonary EMT. As expected, FMT with RA feces suppressed the activated TLR4/NF-κB signaling (Fig. 5F). Consistently, FMT with RA feces markedly reduced the levels of key pro-inflammatory cytokines (TNF-α, IL-1β, IL-6) driven by this pathway (Fig. 5G-I). Moreover, transplantation of feces from RA mice inhibited pulmonary EMT in the hyperoxia-exposed mice, characterized by significantly increased levels of SPC, AQP5, and E-cadherin, while decreased expression of N-cadherin, vimentin and α-SMA (Fig. 5J-L). These results suggested that gut dysbiosis may impair lung development, possibly by activating TLR4/NF-κB signaling and driving pathological EMT.

**Fig. 6:**
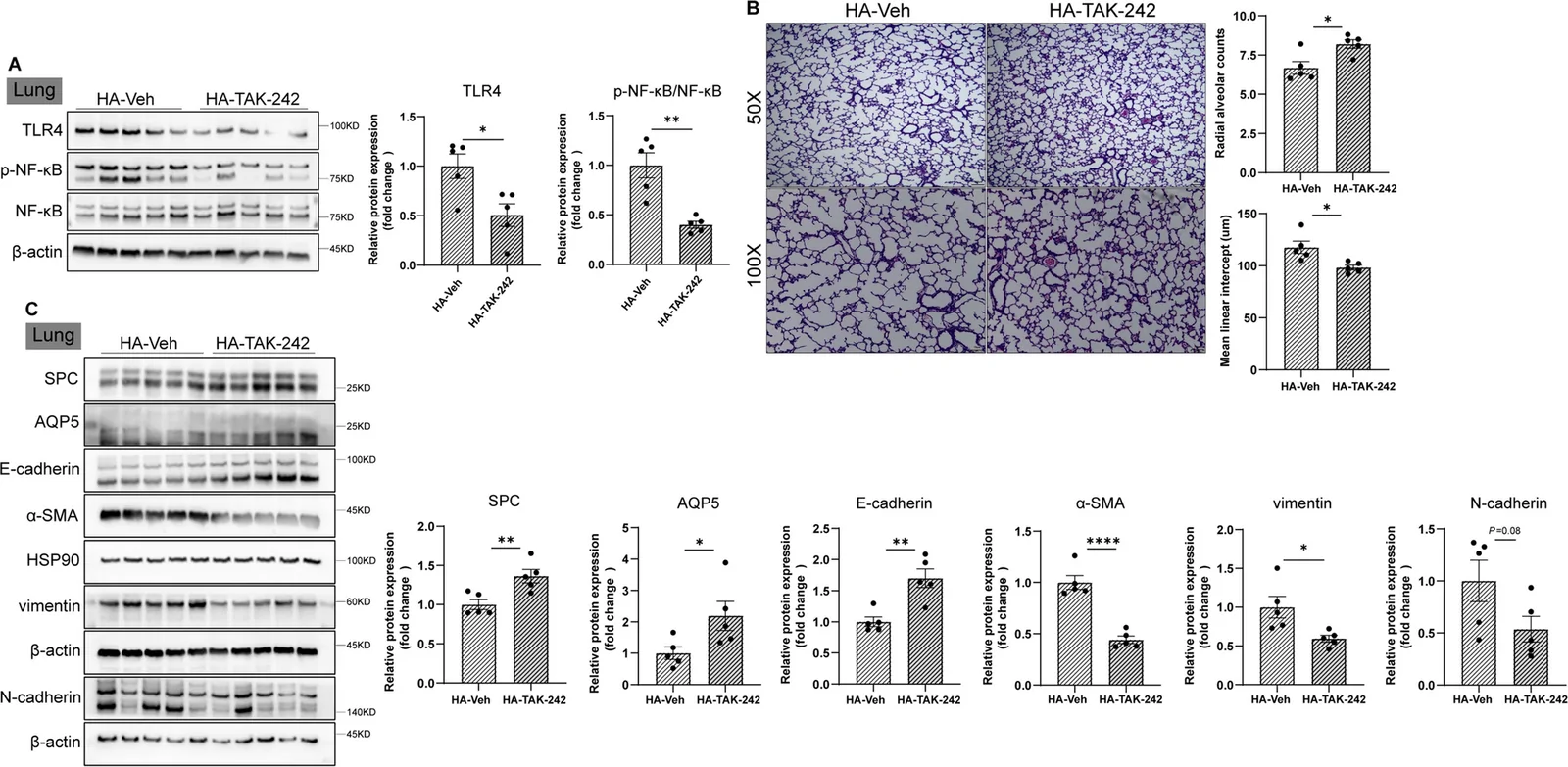
TLR4 inhibitor TAK-242 attenuated pulmonary EMT in hyperoxia-exposed mice. **A** Immunoblot analysis of TLR4 and p-NF-κB p65 in lung tissues of HA-Veh and HA-TAK-242 mice. **B** Representative lung histology, RAC and MLI in HA-Veh and HA-TAK-242 mice (scale bar, 250 μm and 75 μm). **C** Representative western blots and quantification of SPC, AQP5, E-cadherin, vimentin, N-cadherin, and α-SMA in lung tissues of HA-Veh and HA-TAK-242 mice. Data were presented as mean ± SEM and representative of *n* = 5 mice per experimental group. Data are analyzed using Student’s *t*-test. ^*^P < 0.05, ^**^P < 0.01, and ^****^P < 0.0001 vs. the HA-Veh group

To further examine the role of the TLR4/NF-κB pathway in pulmonary EMT during BPD development, we utilized a TLR4 inhibitor (TAK-242). As shown in Fig. 6A, TAK-242 treatment significantly inhibited the upregulated expression of TLR4 and NF-κB p65 phosphorylation in the lungs of hyperoxia-induced BPD mice. Furthermore, TAK-242 treatment ameliorated hyperoxia-induced alveolar simplification, as demonstrated by increased RAC and decreased MLI (Fig. 6B). Importantly, TAK-242 treatment recapitulated the beneficial effects of FMT on EMT markers, upregulating the suppressed levels of SPC, AQP5, and E-cadherin, and downregulating the elevated levels of N-cadherin, vimentin and α-SMA in the BPD group (Fig. 6C). Taken together, these findings suggested that gut dysbiosis impaired lung development in hyperoxia-induced BPD mice. This detrimental effect was mediated, at least partially, through the activation of the TLR4/NF-κB signaling pathway, which subsequently promoted pulmonary EMT. The concordant results from FMT and TLR4 inhibitor (TAK-242) strongly supported this mechanistic link between gut microbiota dysbiosis and TLR4/NF-κB-driven pulmonary EMT in BPD pathogenesis.

## Discussion

**Fig. 7:**
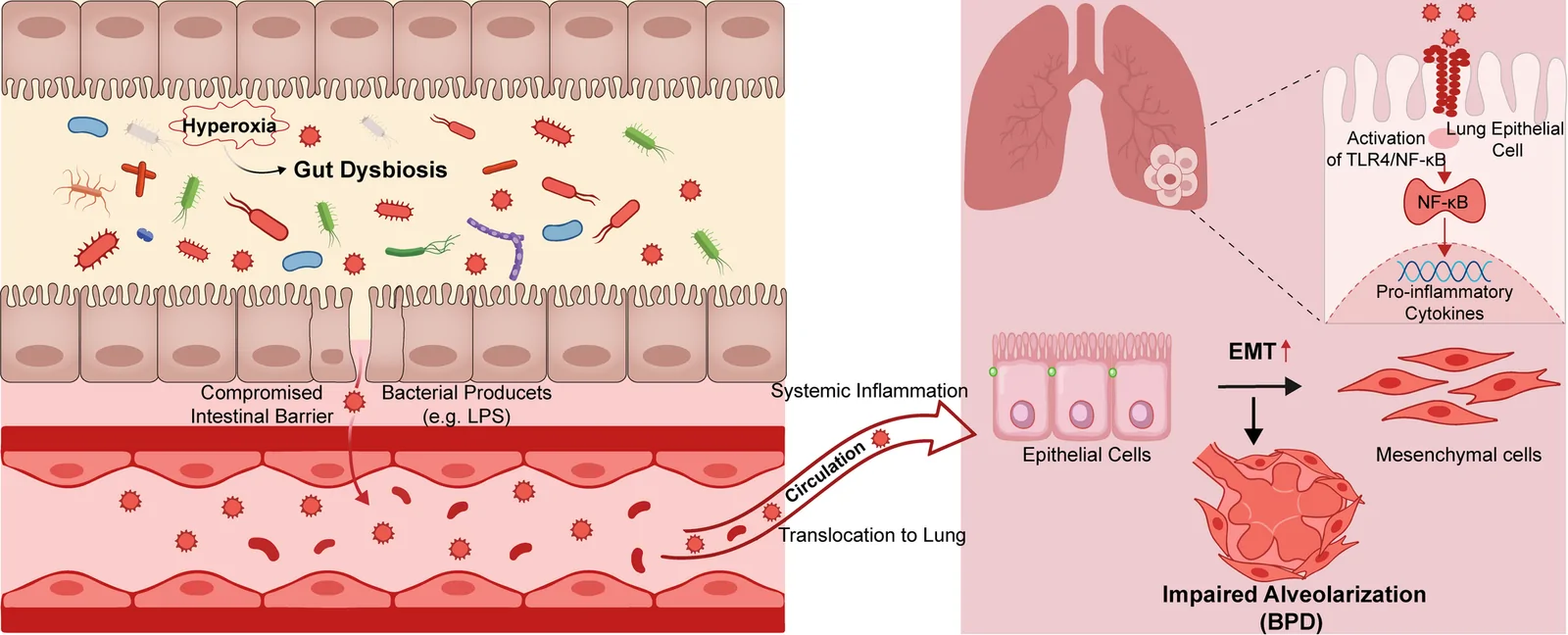
Schematic diagram for this study. Bidirectional communication exists between the lung and the gut, mediated in part by gut microbiota-derived components, particularly LPS from the outer membrane of Gram-negative bacteria. Bronchopulmonary dysplasia caused by hyperoxia induces gut dysbiosis, accompanied with increased LPS, which activates the TLR4/NF-κB inflammatory pathway, thereby promoting EMT in the lung, and ultimately inducing impaired alveolarization

In this study, hyperoxia-induced BPD mice showed reduced alveolarization with increased lung inflammation. Gut microbiota dysbiosis, gut barrier dysfunction, activated lung TLR4/NF-κB signaling, and enhanced lung EMT were also observed in BPD mice compared to the RA mice. These changes were exacerbated by early postnatal antibiotics exposure, whereas transplantation of gut microbiota from RA mice ameliorated these changes. Furthermore, TLR4 inhibitor (TAK-242) significantly diminished hyperoxia-induced EMT process. This study offers new insight into the mechanisms underlying hyperoxia-induced lung developmental impairment, highlighting a role for microbiota dysbiosis and pulmonary TLR4/NF-κB-mediated EMT (Fig. 7).

The gut microbiota has been reported to exert distal effects on the host, such as lung, a phenomenon also termed as the gut-lung axis (Özçam and Lynch 2024; Gallacher et al. 2020; Budden et al. 2017). Recent advances in microbiomics and metagenomics have deepened our understanding of the gut-lung axis. Dysbiosis, characterized by disruptions in the composition and functionality of the gut microbiota, plays a pivotal role in modulating host immune responses and has been implicated in the pathogenesis of various pulmonary diseases, such as chronic obstructive pulmonary disease (COPD), asthma, and acute lung injury (ALI) (Wypych et al. 2019; Wang et al. 2023). Clinical studies in preterm infants have reported altered gut microbial diversity and enrichment of potentially pro-inflammatory taxa in association with BPD risk, suggesting that intestinal dysbiosis may emerge early in disease evolution (Gallacher et al. 2020; Ryan et al. 2019; Zhang et al. 2022; Thatrimontrichai et al. 2025; Ning et al. 2025). However, the biological significance of these associations has remained uncertain. Our data provide experimental confirmation of the idea that gut microbial dysbiosis may influence the immature lung through systemic mechanisms, particularly those involving gut barrier failure and pulmonary inflammation. This is especially plausible in preterm neonates, whose intestinal and pulmonary barriers are both structurally and immunologically immature and therefore likely more vulnerable to environmental perturbations (Lemme et al. 2022; Sampah and Hackam 2020; Arboleya et al. 2021). At the same time, hyperoxia exerts direct toxic effects on the immature lung, including oxidative stress, mitochondrial injury, DNA damage, endothelial dysfunction, altered growth factor signaling, and disruption of alveolar epithelial differentiation (Zhu et al. 2025; Vila Ellis et al. 2025; Ambalavanan et al. 2026). These mechanisms have been extensively implicated in impaired septation and vascular development. Therefore, gut dysbiosis is unlikely to be the sole factor responsible for the hyperoxia‑induced BPD phenotype. Rather, our results suggest that gut microbial dysbiosis may amplify or sustain hyperoxia-induced alveolar simplification and pulmonary inflammation.

One of the most biologically coherent observations in this study is the parallel occurrence of gut barrier dysfunction and pulmonary innate immune activation. The intestinal epithelium provides a critical defense against luminal pathogens and endotoxins (Tang et al. 2021; Martel et al. 2022). Disruption of the gut microbiota compromises this barrier, leading to increased systemic release of inflammatory mediators that promote lung inflammation, alter the pulmonary microenvironment, and exacerbate BPD (Gao et al. 2024; Zhu and Ding 2025). Consistent with the findings of Shen et al. (Shen et al. 2024), in our model, hyperoxia reduced the expression of colonic tight junction proteins and increased the levels of LPS in serum and lung, consistent with translocation of microbial components into the circulation. This is mechanistically relevant because LPS is a well-established ligand for TLR4, and excessive TLR4 activation has been implicated in lung injury (Luo et al. 2025; Tang et al. 2021). Previous studies have shown that aberrant TLR4 signaling can promote inflammatory cytokine production, interfere with normal airway branching and alveolarization, and exacerbate tissue injury in the immature lung (Wedgwood et al. 2020). Our findings that hyperoxia promoted pulmonary TLR4/NF-κB activation and that TLR4 inhibition attenuated lung injury are therefore consistent with our model in which gut barrier dysfunction contributes to distal inflammatory programming of the lung.

Nevertheless, the TLR4/NF-κB axis should not be considered the sole pathway linking gut dysbiosis to BPD. Several complementary mechanisms warrant consideration. First, microbiota-derived metabolites, including short-chain fatty acids, bile acid derivatives, and tryptophan metabolites, can modulate systemic immunity, epithelial differentiation, and inflammatory tone (Yi et al. 2026; Chen et al. 2025). Second, dysbiosis may alter immune cell trafficking or maturation, including macrophage polarization, neutrophil recruitment, and T-cell differentiation, all of which are relevant to neonatal lung injury (Yi et al. 2026; Chen et al. 2025). Third, the gut microbiota could modulate oxidative stress responses and antioxidant defenses, thereby modifying the susceptibility of the developing lung to hyperoxia (Zhang et al. 2024; Freeman et al. 2023). Our current study was not designed to dissect these alternative pathways, and therefore our mechanistic conclusions should be interpreted with caution.

EMT has been implicated in tissue remodeling and fibrosis across multiple organs (Xuan et al. 2023; Macias-Ceja et al. 2023), including the lung, and prior studies suggest that hyperoxia can induce EMT-like changes in alveolar epithelial cells in BPD models (Hua et al. 2025; Yang et al. 2014; Hou et al. 2015). Aberrant activation of EMT promotes the transdifferentiation of epithelial cells into fibroblast-like cells, leading to altered mechanical properties within the alveolar microenvironment and disruption of alveolar formation and secondary septation (2019; Bartis et al. 2014; Jolly et al. 2018). Our data show that hyperoxia increased EMT-related alterations and that both FMT and TLR4 inhibition attenuated this response, suggesting that EMT may be a downstream consequence of inflammatory response and innate immune signaling triggered by gut dysbiosis. However, EMT in developmental lung injury should also be interpreted cautiously. The EMT process occurs along a continuum, and molecular markers alone may not always effectively distinguish complete transdifferentiation from partial epithelial activation or maladaptive repair (Lamouille et al. 2014). Therefore, while our results highlight the involvement of EMT-related remodeling, they do not establish EMT as the exclusive or dominant cause of arrested alveolarization. Interactions with other pathways, especially TGF-β signaling, extracellular matrix remodeling, and fibroblast activation, are likely to be important and deserve further investigation (Lamouille et al. 2014).

The antibiotic experiments further support the importance of gut microbial homeostasis, but they also represent a key limitation. Antibiotics are the most prescribed class of medications in neonatal intensive care units (NICUs) (Ting et al. 2016), and epidemiologic studies have linked prolonged early postnatal antibiotics exposure with adverse outcomes, including increased risk of BPD (Rina et al. 2020; Cantey et al. 2016, 2018; Ran et al. 2021; Shi et al. 2024). In our study, antibiotic treatment aggravated hyperoxia-induced dysbiosis, gut barrier disruption, endotoxemia, inflammatory signaling, and lung injury. These findings are consistent with the possibility that disruption of early microbial colonization increases vulnerability to developmental lung injury. However, antibiotics are inherently non-specific and may exert direct effects on host tissues independent of microbiota. They may influence epithelial metabolism, immune maturation, or mitochondrial function, all of which could confound causal interpretation (Morgun et al. 2015). Therefore, the antibiotic model should be regarded as supportive but not definitive evidence for the role of dysbiosis. The FMT rescue experiments strengthen the argument for microbial involvement, but they do not fully eliminate the possibility of antibiotic-independent effects.

Several limitations of this study merit consideration. First, the antibiotics regimen employed in this study are those commonly applied in animal research and may not fully recapitulate the spectrum, timing, or dosing of antibiotics administered to preterm infants during the early postnatal period. Second, while BPD maybe more frequent among males than females, potential sex-specific differences were not addressed in the animal models used in this study. Third, although FMT improved the phenotype, 16S rRNA sequencing remains limited in resolution. Therefore, we cannot conclude with certainty which microbial taxa or functions mediated the protective effects of FMT. Future studies should systematically investigate the effects of specific microbial interventions and microbiota-derived metabolites to elucidate the precise mechanisms and therapeutic potential of targeted gut microbiota modulation in BPD.

## Conclusion

In summary, our findings indicate that hyperoxia induces BPD by disrupting the gut microbiota, leading to intestinal barrier dysfunction, heightened pulmonary inflammation and epithelial-mesenchymal transition via activation of TLR4/NF-κB pathway. Antibiotic exposure further amplifies these deleterious effects. These findings establish a mechanistic link between gut dysbiosis and impaired lung development, positioning the gut-lung axis as a critical contributor to BPD pathogenesis and a potential therapeutic target. It also highlights the importance of minimizing unnecessary antibiotic use and preservation of gut microbial homeostasis.

## Supplementary Information

Additional file 1. Figure S1. Early postnatal administration of antibiotics did not impair pulmonary development in normoxic mice.

Additional file 2. Figure S2. Early-postnatal antibiotics treatment promoted the TLR4/NF-κB signaling overactivation and EMT in the lung of hyperoxia-exposed mice.

Additional file 3. Figure S3. The results of 16S rRNA sequencing.

Additional file 4. Table S1. Primers for qRT-PCR.

Additional file 5. The ethics documents for this study.

Additional file 6. Uncropped Gels and Blots images.

## Data Availability

All data are available from the corresponding authors upon reasonable request.

## Abbreviations

BPD: Bronchopulmonary dysplasia
EMT: Epithelial-mesenchymal transition
FMT: Fecal microbiota transplantation
TLR4: Toll-like receptor 4
NF-κB: Nuclear factor kappa-B
RA: Room air
ABX: Antibiotic treatment
GF: Germ-free
MLI: Mean linear intercept
RAC: Radial alveolar count
HA-ABX: ABX‐treated hyperoxia-exposed
HA-PBS: PBS‐treated hyperoxia-exposed
LPS: Lipopolysaccharide
PCoA: Principal coordinate analysis
LEfSe: Linear discriminant analysis effect size
AEC: Alveolar epithelial cells
TNF-α: Tumor necrosis factor-α
IL-1β: Interleukin-1β
IL-6: Interleukin-6
NICU: Neonatal intensive care unit
HE: Hematoxylin and eosin
