# Supplementary material for: Gut dysbiosis modulates hyperoxia-induced bronchopulmonary dysplasia by promoting EMT through activating TLR4/NF-κB pathway
Source: Mol Med. 2026 Jun 16;32:135. doi: 10.1186/s10020-026-01529-x (PMC13508397; doi:10.1186/s10020-026-01529-x)
Supplement: Supplementary file 1 [file 10020_2026_1529_MOESM5_ESM.pdf]

(NO.2024050713314283)

广州市妇女儿童医疗中心实验动物福利伦理审查表
(课题申报)

Experimental Animal Welfare and Ethics Review Form of Guangzhou Women and Children' s Medical Center (Application for Entry into Experimental Animal Facility)

动物伦理审查编号(No): KTDW-2024-00973

课题申报依托单位<br>Project Application Supporting Institution	广州市妇女儿童医疗中心		
项目负责人<br>Project Leader	颜亚琴	联系电话<br>Telephone	15626212664
课题名称<br>Project title	生后早期抗生素暴露对新生小鼠BPD发生发展的作用和机制研究		
课题类型<br>Type of Project	自选课题		
实验动物设施名称、地点及编号	广州市妇女儿童医疗中心实验动物中心(白云区新达路91,93号)9楼，编号:SYXK(粤)2023-0320		
动物种类、品系及数量<br>Variety,Strain, and QuantitySpecies ofStrain	C57BL/6J-SPF-50		
动物实验起止日期<br>Animal Experiment Dates	2024-05-20	至 2025-05-31	
项目负责人声明<br>Statement by theProject Leader	我承诺遵守动物福利伦理，接受监督检查，若违规将自愿受罚。<br>I pledge to adhere to animal welfare ethics, undergo oversight, and accept penalties for anyviolations.<br>项目负责人签名(手签): 颜亚琴<br>申请时间: 2024年 5月24日		
实验动物福利伦理委员会审查意见<br>Review Opinion of the LaboratoryAnimal Welfare and EthicsCommittee	该实验方案符合动物福利伦理要求，通过动物实验伦理审查准予开展动物实验。<br>The experiment plan meets ethicalstandards and is authorized for animal testing.	委员会主席签名(手签印)<br>Chairman's Hand-signed Signature<br>[seal]	
备注<br>Remark	实验动物福利伦理委员会签章<br>Seal of the Laboratory Animal Welfare and Ethics Committee<br>(广州市妇女儿童医疗中心 实验动物福利伦理委员会)		

CS 扫描全能王
3亿人都在用的扫描App
