# Supplementary material for: Gut dysbiosis modulates hyperoxia-induced bronchopulmonary dysplasia by promoting EMT through activating TLR4/NF-κB pathway
Source: Mol Med. 2026 Jun 16;32:135. doi: 10.1186/s10020-026-01529-x (PMC13508397; doi:10.1186/s10020-026-01529-x)
Supplement: Supplementary file 2 [file 10020_2026_1529_MOESM6_ESM.docx]

Fig.1B

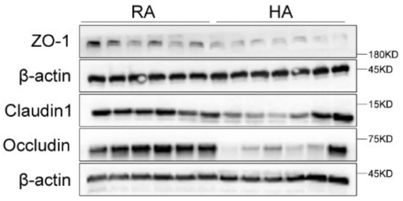
RA
HA
ZO-1
β-actin
Claudin1
Occludin
β-actin
180KD
45KD
15KD
75KD
45KD

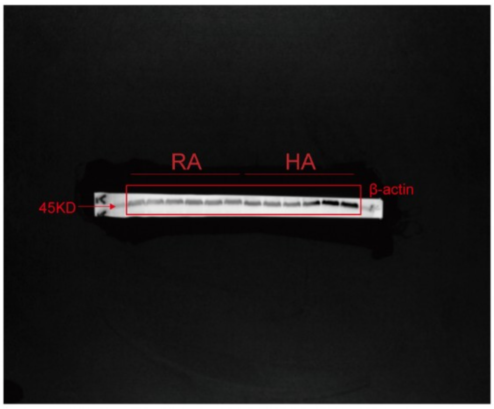
RA
HA
β-actin
45KD

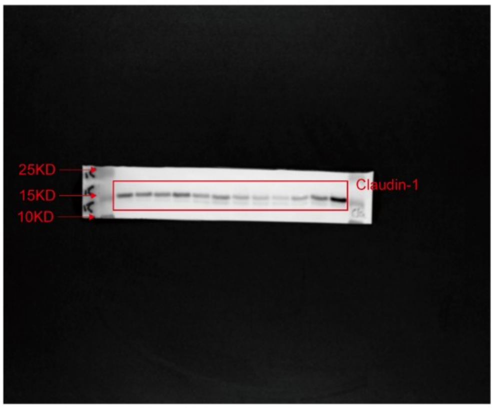
25KD
15KD
10KD
Claudin-1

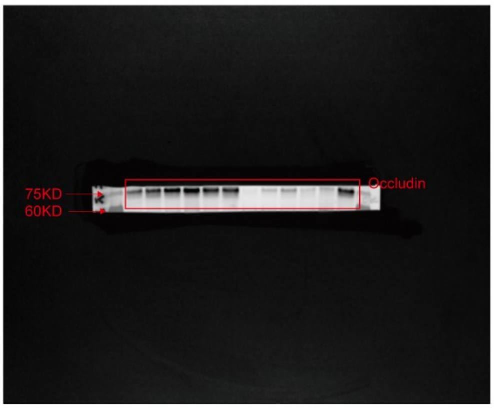
Occludin
75KD
60KD

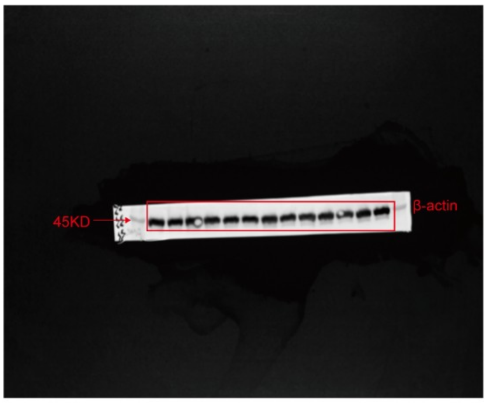
β-actin
45KD

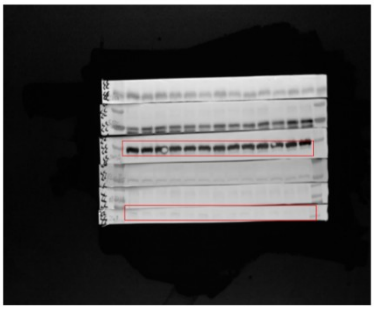

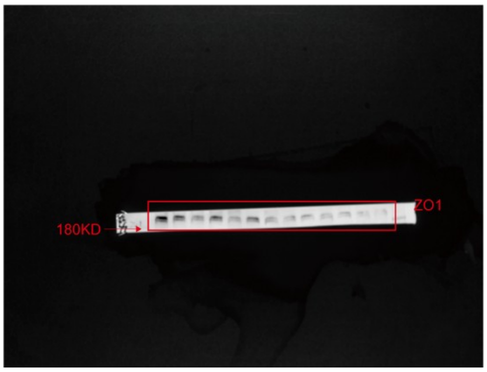
ZO1
180KD

Fig.2B

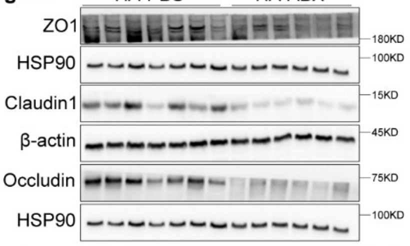
HA-PBS
HA-ABX
ZO1
HSP90
Claudin1
β-actin
Occludin
HSP90
180KD
100KD
15KD
45KD
75KD
100KD

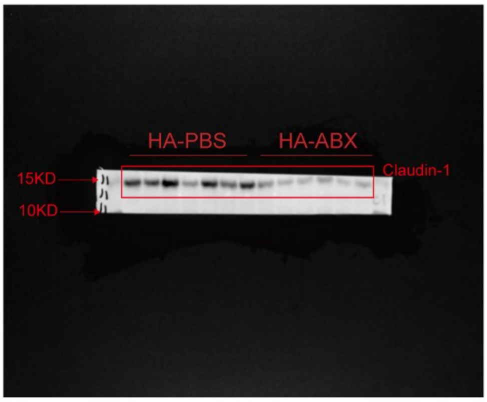
HA-PBS
HA-ABX
15KD
10KD
Claudin-1

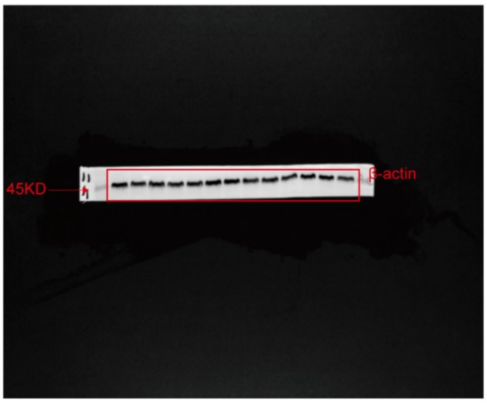
45KD
β-actin

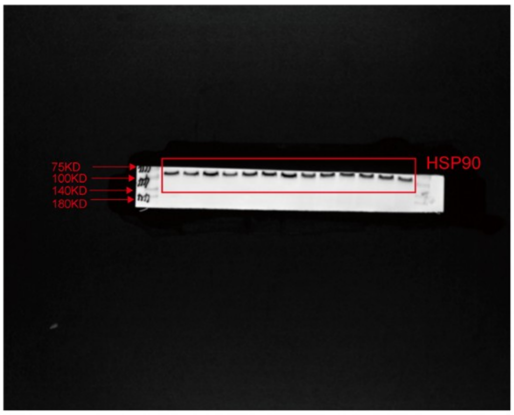
75KD
100KD
140KD
180KD
HSP90

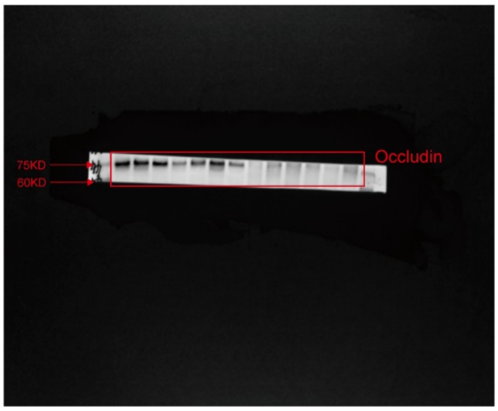
75KD
60KD
Occludin

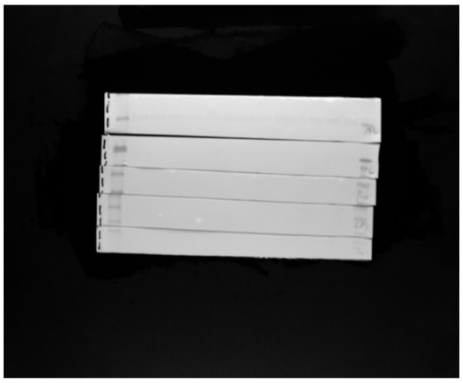

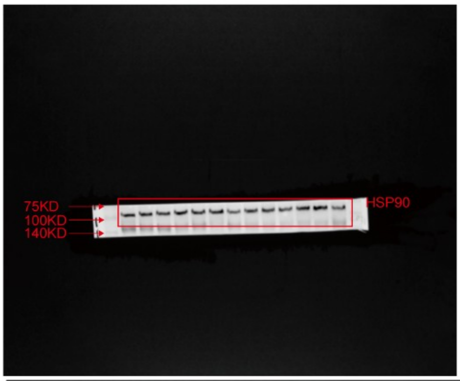
75KD
100KD
140KD
HSP90

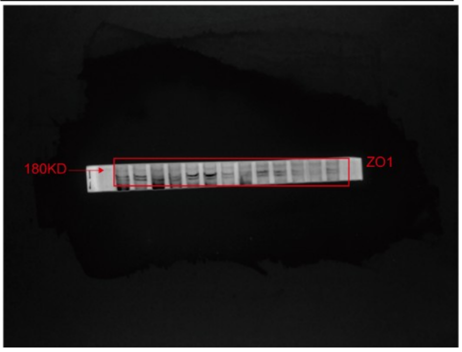
180KD
ZO1

Fig.4A

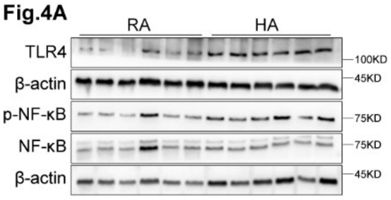
RA
HA
TLR4
β-actin
p-NF-κB
NF-κB
β-actin
100KD
45KD
75KD
75KD
45KD

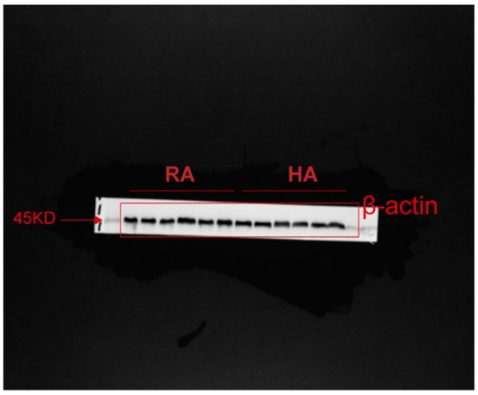
RA
HA
45KD
β-actin

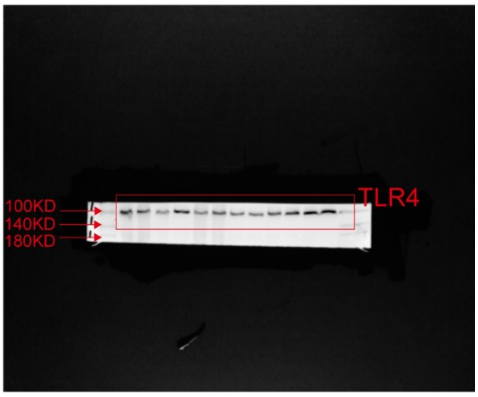
100KD
140KD
180KD
TLR4

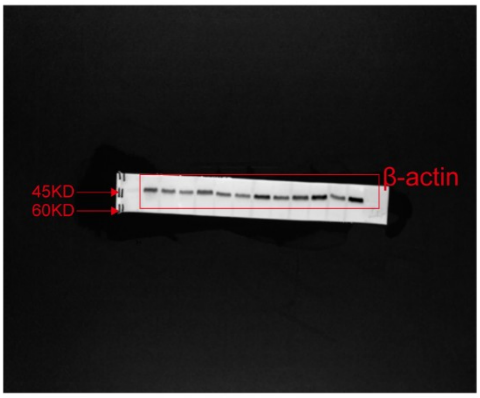
45KD
60KD
β-actin

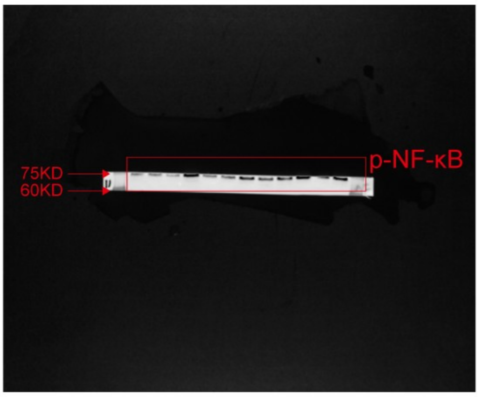
75KD
60KD
p-NF-κB

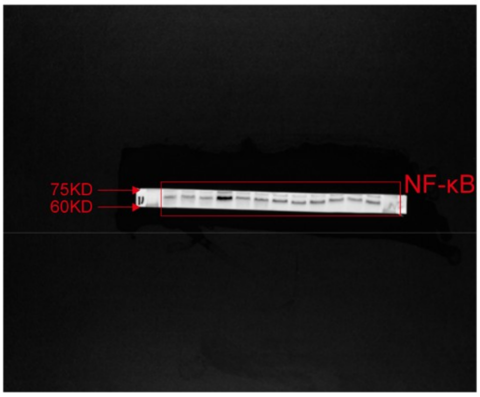
75KD
60KD
NF-κB

Fig.4E

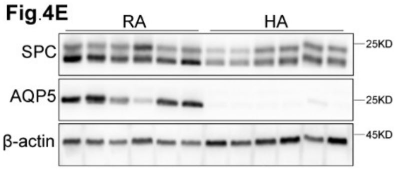
RA
HA
SPC
AQP5
β-actin
25KD
25KD
45KD

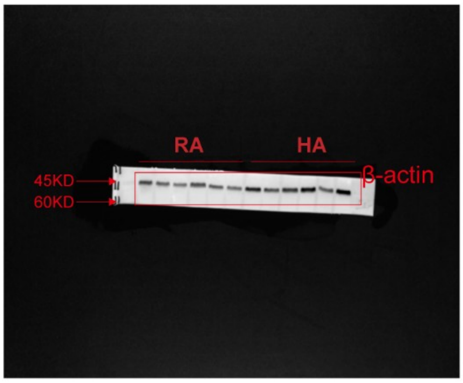
RA
HA
45KD
60KD
β-actin

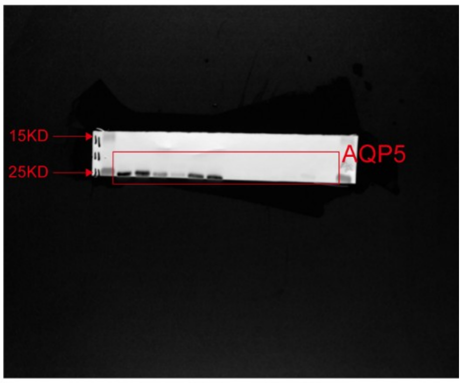
15KD
25KD
AQP5

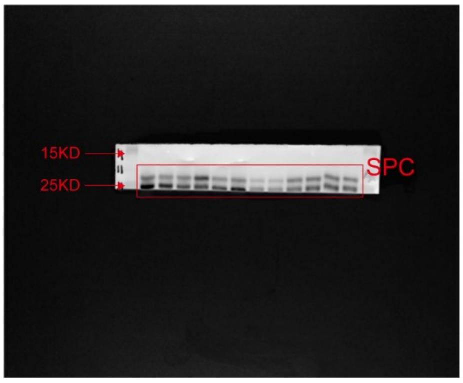
15KD
25KD
SPC

Fig. 4F

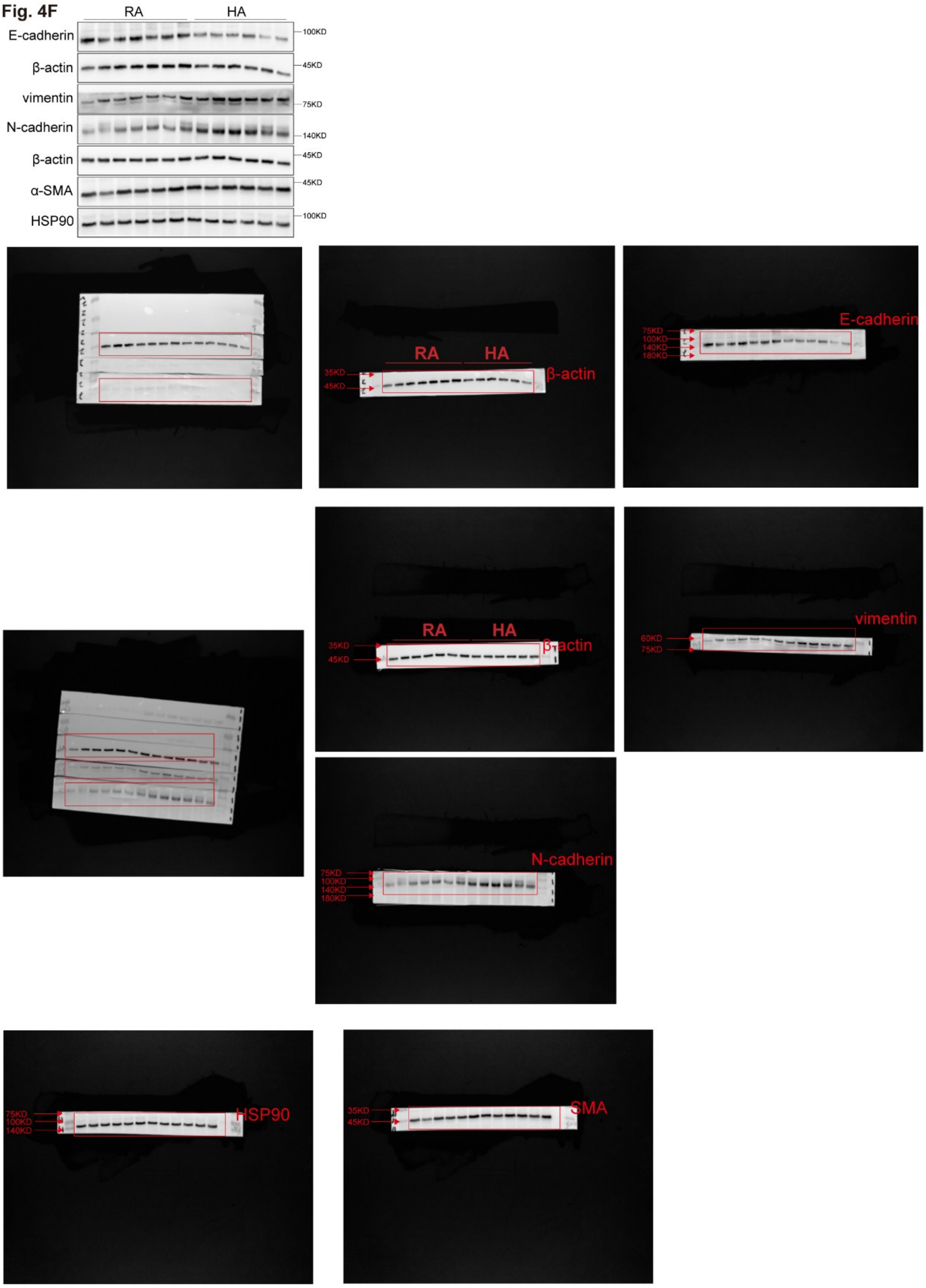

RA
HA
E-cadherin
β-actin
vimentin
N-cadherin
β-actin
α-SMA
HSP90
100KD
45KD
75KD
140KD
45KD
45KD
100KD
RA
HA
β-actin
35KD
45KD
E-cadherin
75KD
100KD
140KD
180KD
RA
HA
β-actin
35KD
45KD
vimentin
60KD
75KD
N-cadherin
75KD
100KD
140KD
180KD
HSP90
75KD
100KD
140KD
SMA
35KD
45KD

Fig. S2A

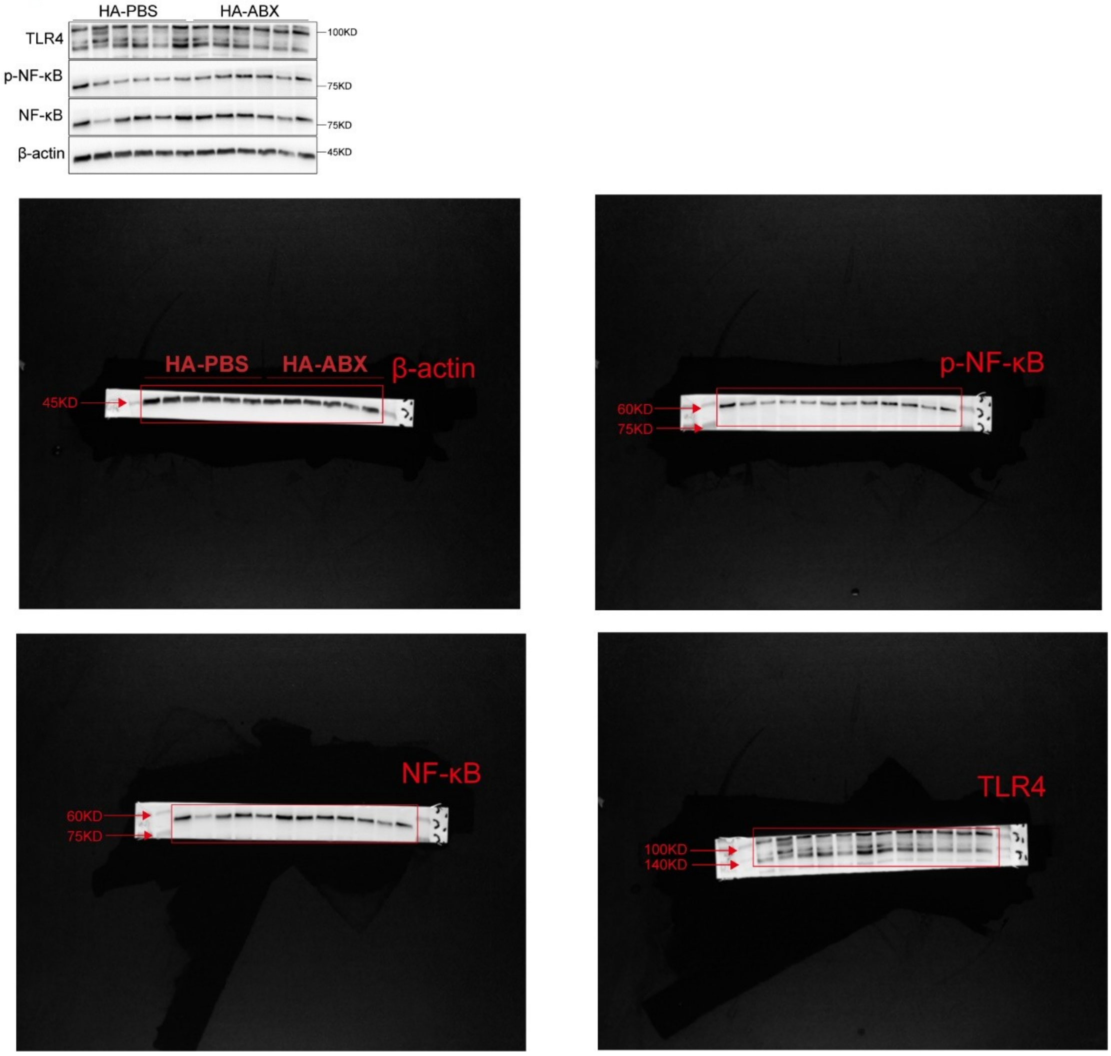
HA-PBS
HA-ABX
TLR4
p-NF-κB
NF-κB
β-actin
100KD
75KD
75KD
45KD
HA-PBS
HA-ABX
β-actin
45KD
p-NF-κB
60KD
75KD
NF-κB
60KD
75KD
TLR4
100KD
140KD

Fig. S2E

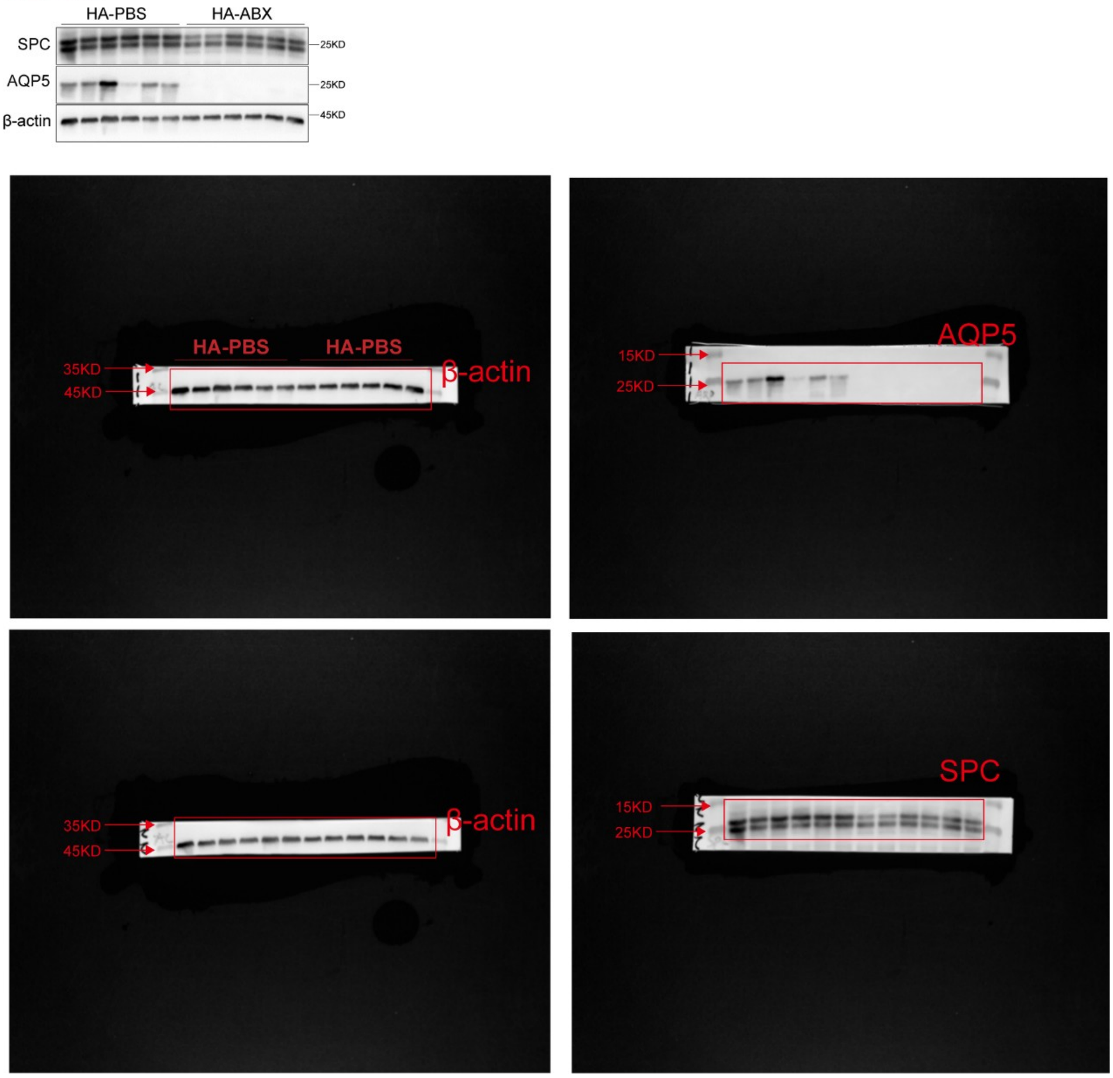
HA-PBS
HA-ABX
SPC
AQP5
β-actin
25KD
25KD
45KD
HA-PBS
HA-PBS
35KD
45KD
β-actin
AQP5
15KD
25KD
35KD
45KD
β-actin
SPC
15KD
25KD

Fig. S2F

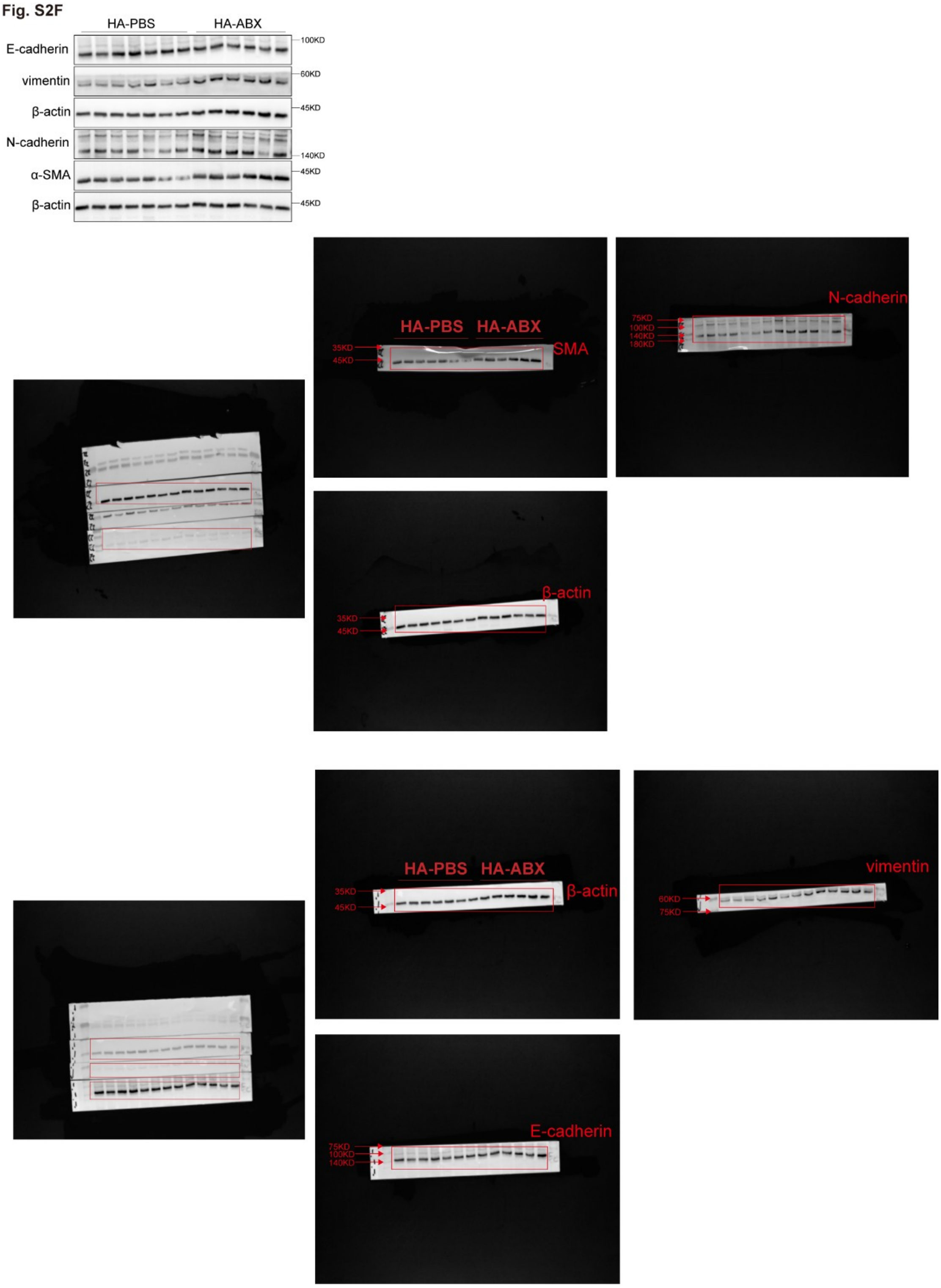
HA-PBS
HA-ABX
E-cadherin
vimentin
β-actin
N-cadherin
α-SMA
β-actin
100KD
60KD
45KD
140KD
45KD
45KD
HA-PBS
HA-ABX
SMA
35KD
45KD
N-cadherin
75KD
100KD
140KD
180KD
β-actin
35KD
45KD
HA-PBS
HA-ABX
β-actin
35KD
45KD
vimentin
60KD
75KD
E-cadherin
75KD
100KD
140KD

Fig. 5B

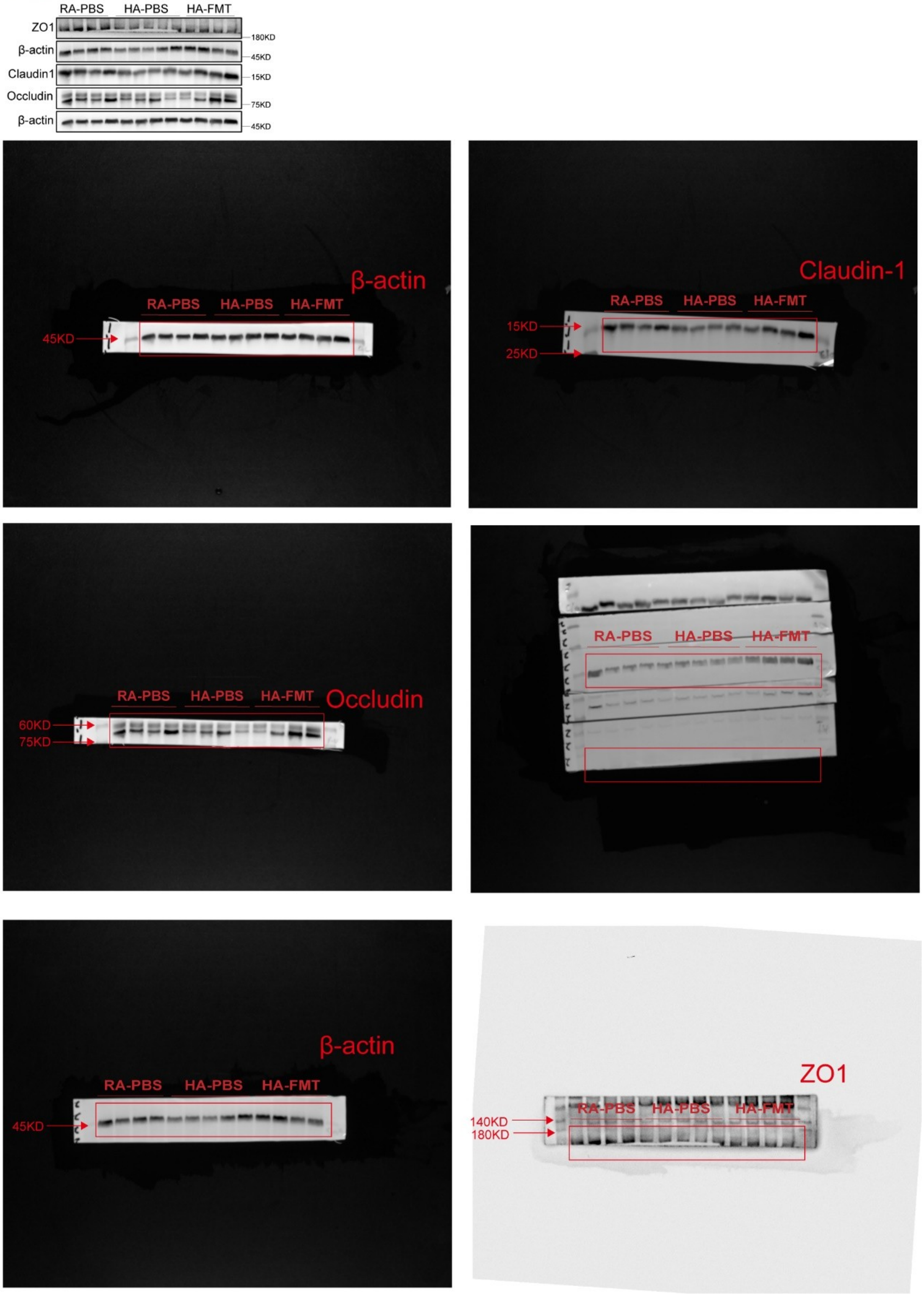
RA-PBS
HA-PBS
HA-FMT
ZO1
β-actin
Claudin1
Occludin
β-actin
180KD
45KD
15KD
75KD
45KD
β-actin
RA-PBS
HA-PBS
HA-FMT
45KD
Claudin-1
RA-PBS
HA-PBS
HA-FMT
15KD
25KD
RA-PBS
HA-PBS
HA-FMT
Occludin
60KD
75KD
RA-PBS
HA-PBS
HA-FMT
β-actin
RA-PBS
HA-PBS
HA-FMT
45KD
ZO1
RA-PBS
HA-PBS
HA-FMT
140KD
180KD

Fig. 5F

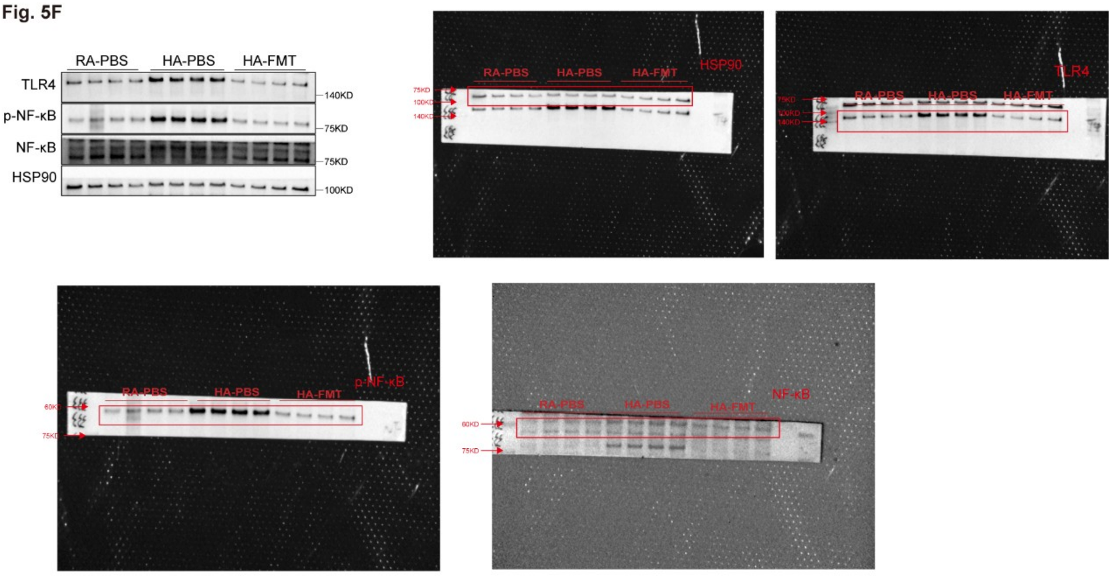
RA-PBS
HA-PBS
HA-FMT
TLR4
p-NF-κB
NF-κB
HSP90
140KD
75KD
75KD
100KD
HSP90
TLR4
p-NF-κB
NF-κB

Fig. 5J

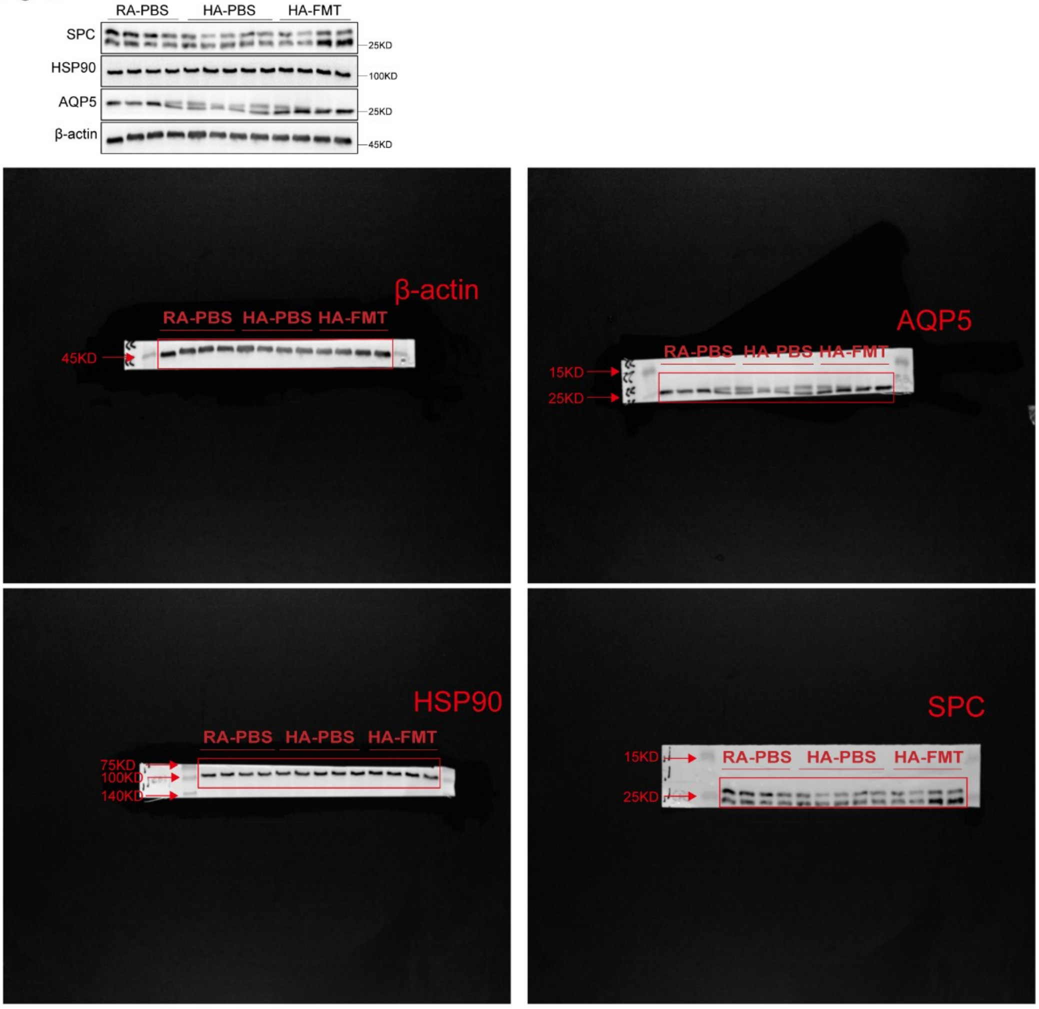
RA-PBS
HA-PBS
HA-FMT
SPC
HSP90
AQP5
β-actin
25KD
100KD
25KD
45KD
β-actin
45KD
AQP5
15KD
25KD
HSP90
75KD
100KD
140KD
SPC
15KD
25KD

Fig. 5L

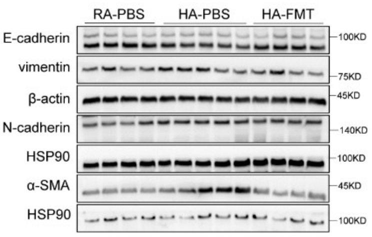
RA-PBS
HA-PBS
HA-FMT
E-cadherin
vimentin
β-actin
N-cadherin
HSP90
α-SMA
HSP90
100KD
75KD
45KD
140KD
100KD
45KD
100KD

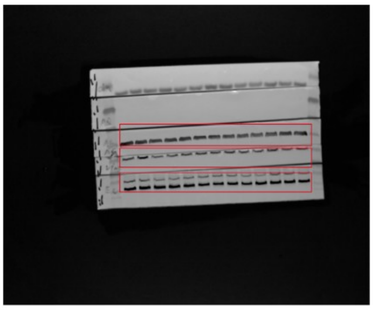

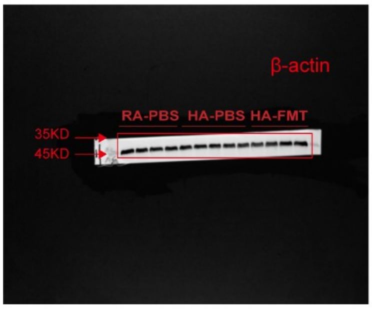
β-actin
RA-PBS HA-PBS HA-FMT
35KD
45KD

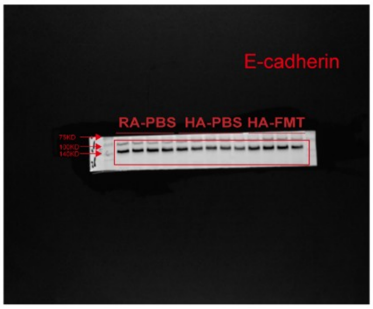
E-cadherin
RA-PBS HA-PBS HA-FMT

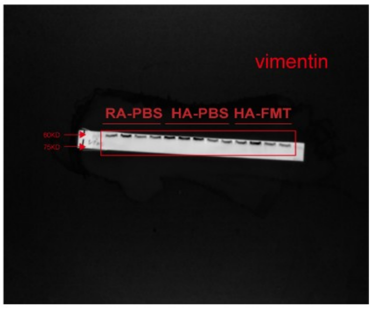
vimentin
RA-PBS HA-PBS HA-FMT

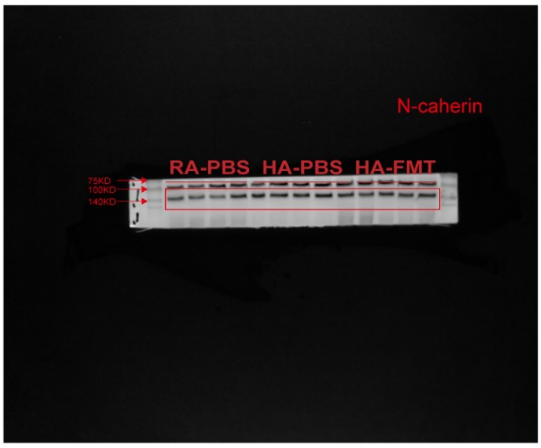
N-caherin
RA-PBS HA-PBS HA-FMT
75KD
100KD
140KD

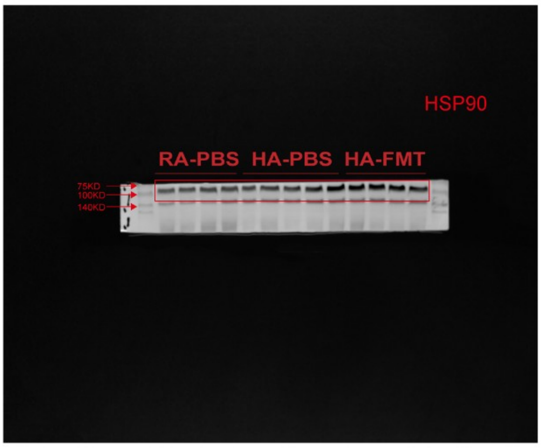
HSP90
RA-PBS HA-PBS HA-FMT
75KD
100KD
140KD

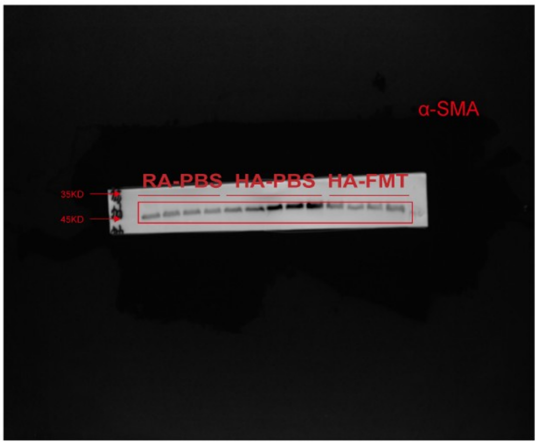
α-SMA
RA-PBS HA-PBS HA-FMT
35KD
45KD

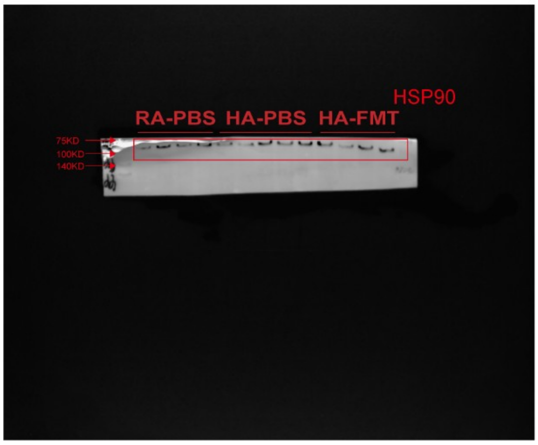
HSP90
RA-PBS HA-PBS HA-FMT
75KD
100KD
140KD

Fig. 6A

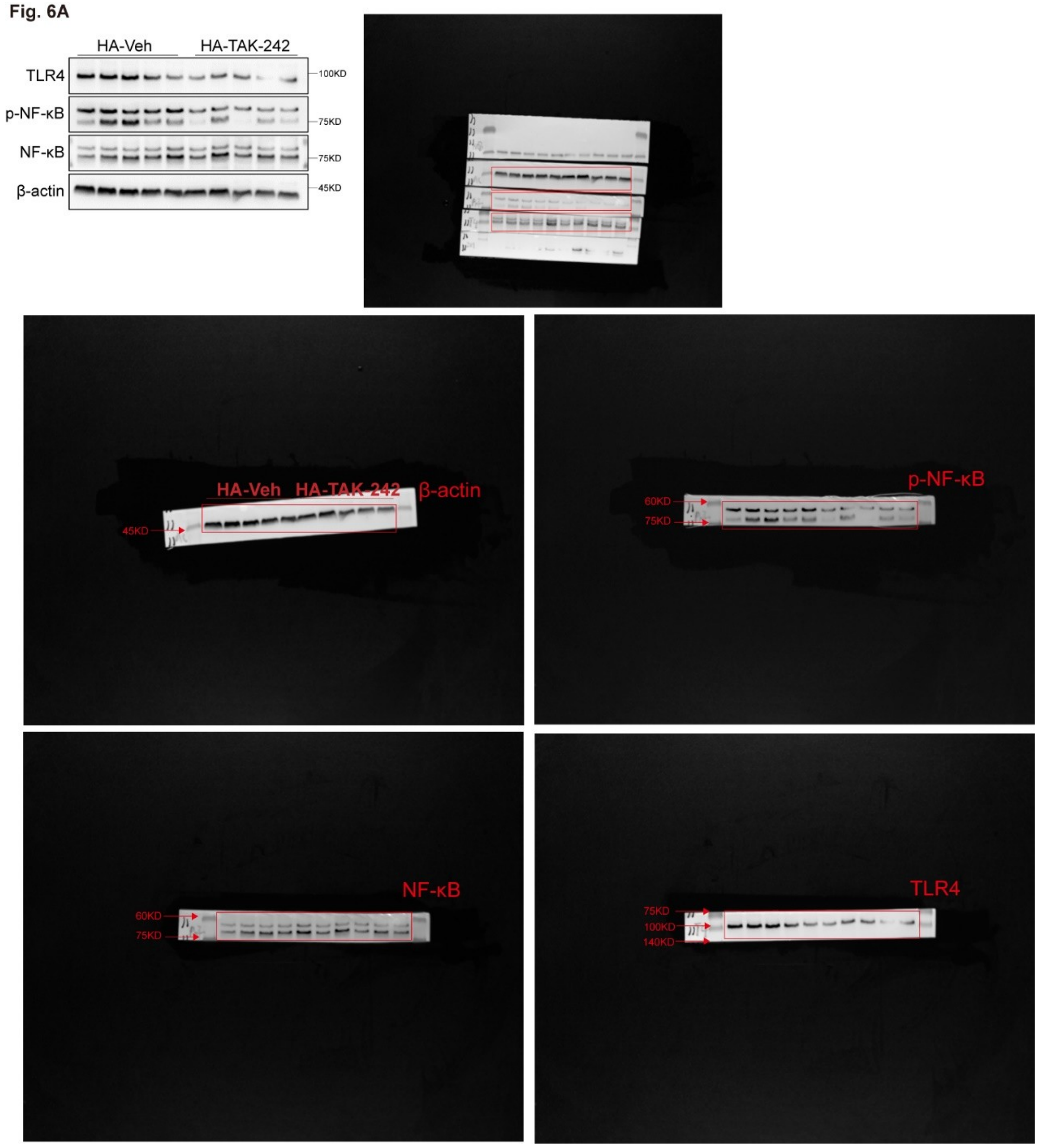
HA-Veh
HA-TAK-242
TLR4
p-NF-κB
NF-κB
β-actin
100KD
75KD
45KD
β-actin
45KD
p-NF-κB
60KD
75KD
NF-κB
60KD
75KD
TLR4
75KD
100KD
140KD

Fig. 6C

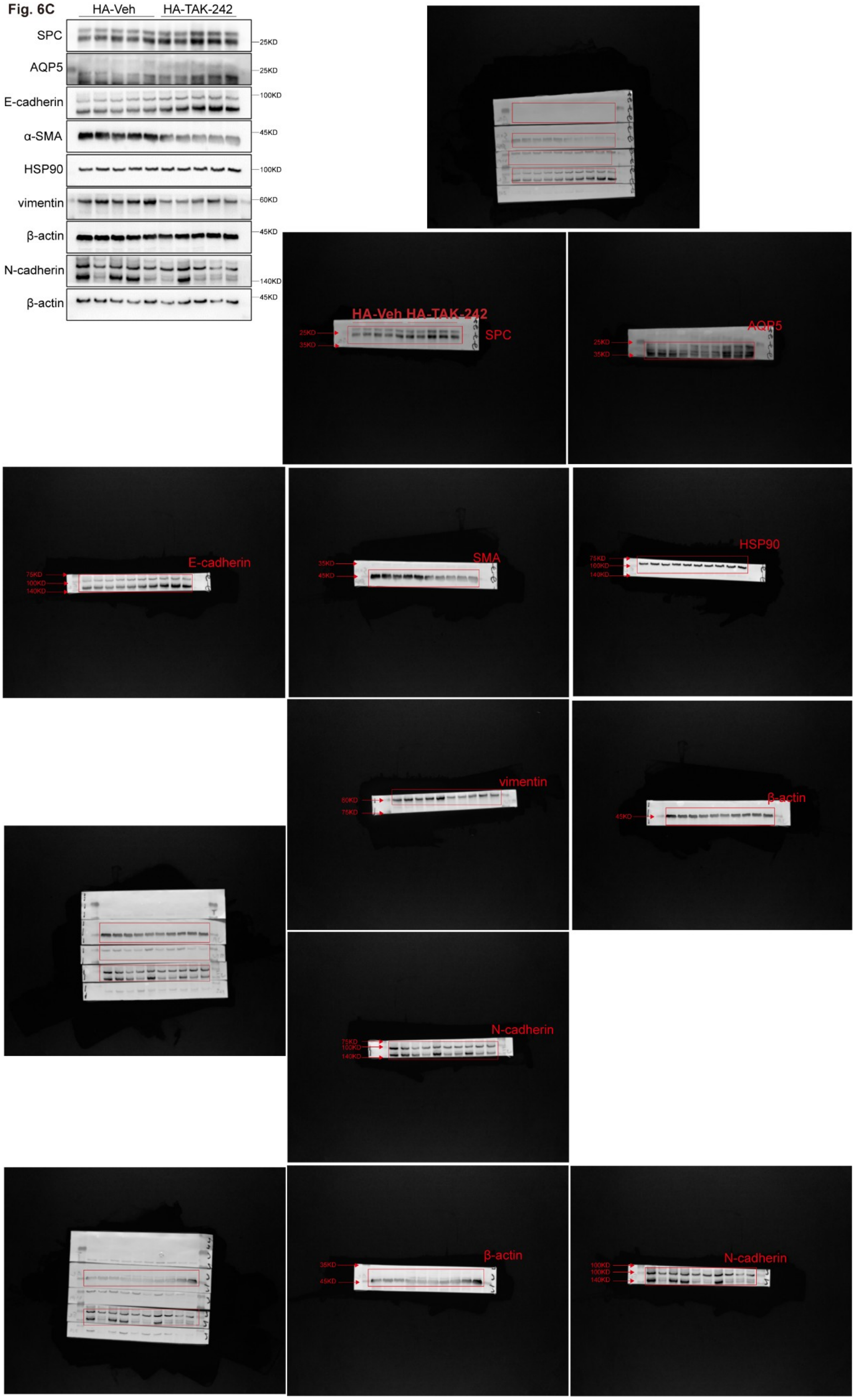

HA-Veh
HA-TAK-242
SPC
AQP5
E-cadherin
α-SMA
HSP90
vimentin
β-actin
N-cadherin
β-actin
25KD
25KD
100KD
45KD
100KD
60KD
45KD
140KD
45KD
HA-Veh HA-TAK-242
SPC
AQP5
E-cadherin
SMA
HSP90
vimentin
β-actin
N-cadherin
β-actin
N-cadherin
