# Supplementary material for: Gut dysbiosis modulates hyperoxia-induced bronchopulmonary dysplasia by promoting EMT through activating TLR4/NF-κB pathway
Source: Mol Med. 2026 Jun 16;32:135. doi: 10.1186/s10020-026-01529-x (PMC13508397; doi:10.1186/s10020-026-01529-x)
Supplement: Supplementary file 2 [file 10020_2026_1529_MOESM1_ESM.docx]

**Fig.S1** Early postnatal administration of antibiotics did not impair pulmonary development in normoxic mice. **(A-C)** Representative lung histology, RAC and MLI in RA-PBS and RA-ABX mice (scale bar, 500 μm and 200 μm).
