# Supplementary material for: Gut dysbiosis modulates hyperoxia-induced bronchopulmonary dysplasia by promoting EMT through activating TLR4/NF-κB pathway
Source: Mol Med. 2026 Jun 16;32:135. doi: 10.1186/s10020-026-01529-x (PMC13508397; doi:10.1186/s10020-026-01529-x)
Supplement: Supplementary file 3 [file 10020_2026_1529_MOESM2_ESM.docx]

**Fig.S2** **Early-postnatal ABX treatment promoted the TLR4/NF-κB signaling overactivation and EMT in the lung of hyperoxia-exposed mice.** **(A)** Representative western blots and quantification of pulmonary TLR4 and p-NF-κB p65 in HA-PBS and HA-ABX mice. **(B)** TNF-α levels in the lung. **(C)** IL-6 levels in the lung. **(D)** IL-1β levels in the lung. (E) Representative western blots and quantification of pulmonary SPC and AQP5 in HA-PBS and HA-ABX mice. **(F)** Immunoblot analysis of E-cadherin, vimentin, N-cadherin, and α-SMA in lung tissues of HA-PBS and HA-ABX mice. Data were presented as mean ± SEM and representative of n=6-8 mice per experimental group. Data are analyzed using Student’s *t*-test **(A-D, F)** or Mann-Whitney U test **(E)**. ^*^*P* < 0.05, ^**^*P* < 0.01, ^***^*P* < 0.001, and ^****^*P* < 0.0001 vs. the HA-PBS group.
