# Supplementary material for: Gut dysbiosis modulates hyperoxia-induced bronchopulmonary dysplasia by promoting EMT through activating TLR4/NF-κB pathway
Source: Mol Med. 2026 Jun 16;32:135. doi: 10.1186/s10020-026-01529-x (PMC13508397; doi:10.1186/s10020-026-01529-x)
Supplement: Supplementary file 4 [file 10020_2026_1529_MOESM3_ESM.docx]

**Fig.S3** **The results of** **16S rRNA sequencing.** **(A-B)** Comparison of Simpson and Shannon of α-diversity index. **(C)** PCoA based on weighted UniFrac distances. **(D)** Bacterial composition at the phylum level. **(E)** Bacterial composition at genus level. n=7-9 mice per experimental group.
