# Supplementary material for: Gut dysbiosis modulates hyperoxia-induced bronchopulmonary dysplasia by promoting EMT through activating TLR4/NF-κB pathway
Source: Mol Med. 2026 Jun 16;32:135. doi: 10.1186/s10020-026-01529-x (PMC13508397; doi:10.1186/s10020-026-01529-x)
Supplement: Supplementary file 5 [file 10020_2026_1529_MOESM4_ESM.docx]

Table S1 Primers for qRT-PCR

| Gene name | Primer sequence (5’-3’) | | Amplicon Length (bp) |
| --- | --- | --- | --- |
| 18S | F | 5’-CGCGGTTCTATTTTGTTGGT-3’ | 150 |
| NR_003278.3 | R | 5’-AGTCGGCATCGTTTATGGTC-3’ |  |
| Zo1 | F | 5’-ACCACCAACCCGAGAAGAC-3’ | 134 |
| NM_009386.3 | R | 5’-CAGGAGTCATGGACGCACA-3’ |  |
| Claudin1 | F | 5’-GGCCTTGGCTGTACCTTACC-3’ | 109 |
| NM_016674.4 | R | 5’-GGAGCACCTTATCCCCGTTT-3’ |  |
| Occludin | F | 5’-CAGGTGAATGGGTCACCGAG-3’ | 170 |
| NM_001360536.1 | R | 5’-CAGGCTCCCAAGATAAGCGA-3’ |  |
